# Shadow‐Calibrated Stereo Vision for Colorimetric Sweat Analysis

**DOI:** 10.1002/advs.75171

**Published:** 2026-04-07

**Authors:** Ting Xiao, Yuwen Yan, Miaorong Lin, Jiahui Chen, Jianxin Meng, Xiang Cui, Peng Zhang, Fengyu Li

**Affiliations:** ^1^ College of Chemistry and Materials Science, Guangdong Provincial Key Laboratory of Speed Capability Research, Su Bingtian Center for Speed Research and Training Jinan University Guangzhou China; ^2^ Department of Orthopedics, National Clinical Research Center for Orthopedics, Sports Medicine & Rehabilitation Chinese PLA General Hospital Beijing China; ^3^ College of Chemistry Zhengzhou University Zhengzhou China

**Keywords:** colorimetric analysis, deep‐learning, shadow‐calibrated, stereo vision, sweat analysis

## Abstract

The image recognition technologies, such as facial recognition and object detection, have found widespread application across diverse fields. However, conventional monocular camera systems capture only 2D information, rendering the accurate reconstruction of 3D morphological features challenging and thereby limiting their utility in precision measurement applications. To address this limitation, this study proposes a novel shadow‐assisted 3D calibration methodology. This approach reconstructs 3D morphology from a monocular camera viewpoint by introducing a controllable light source and leveraging shadow geometry. In this work, we establish a mathematical model for calibrating 3D structures using shadows and employ Convolutional Neural Network‐based 2D image analysis to achieve volumetric calibration. Applied to 3D hydrogel swelling colorimetric analysis for biomarker concentration determination, this method achieves a coefficient of determination (R^2^) exceeding 0.998. The utilization of shadow‐calibrated 3D structural analysis yields a substantial enhancement in analytical accuracy. By design, this “shadow‐calibrated” strategy overcomes the dimensional constraints inherent to traditional 2D colorimetric analysis.

## Graphical abstract

By establishing a mathematical model that reconstructs 3D structures through geometric features of object shadows under controlled illumination, and combining it with Convolutional Neural Network‐based 2D image analysis for volumetric calibration, this work enables highly accurate 3D morphological reconstruction. And applied to 3D gel swelling colorimetric analysis for sweat biomarker detection, the approach significantly reduces systematic quantification biases caused by neglecting swelling‐induced volume effects. The shadow‐calibrated 3D structural analysis achieves a coefficient of determination, demonstrating a substantial enhancement in analytical accuracy.

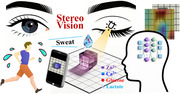

## 1 Introduction

The advancement of artificial intelligence (AI) technology has profoundly reshaped both daily human life and scientific research [1, 2, 3, 4, 5]. AI‐driven image recognition, recognized as a highly valuable and rapidly evolving technology, represents one of the most significant research directions within the field of computer vision [6, 7]. This technology holds substantial application potential across diverse domains, such as security systems [8], medical image diagnostics [9, 10], autonomous driving [11], and facial recognition [12, 13]. The majority of currently employed image capture techniques typically utilize conventional single‐lens cameras, which compress 3D scenes into 2D representations, resulting in the loss of depth information [14]. Currently prevalent methods for measuring 3D information of objects primarily include time‐of‐flight imaging, scanning imaging, structured‐light projection imaging, and stereo vision imaging [15]. While time‐of‐flight imaging and scanning imaging share similarities in employing emitted light sources to measure distance information on object surfaces, they differ in their fundamental principles: time‐of‐flight imaging relies on measuring the round‐trip time of light pulses to calculate distance and acquires data through full‐field parallel acquisition [16], whereas scanning imaging operates via incremental point‐by‐point or line scanning for laser ranging. However, both techniques not only exhibit a strong dependence on costly hardware components and sophisticated software algorithms, but also encounter challenges related to high power consumption and low signal‐to‐noise ratio.

The core mechanism of structured‐light projection imaging involves projecting specific light patterns such as dots, circles, stripes, or encoded structured light onto the object's surface [17]. As the surface geometry varies, these patterns become distorted. A monocular camera captures this distorted pattern, and computational algorithms subsequently derive depth information by quantifying the degree of pattern distortion [18]. This monocular structured‐light scanning technology finds extensive application in high‐precision scanning of small‐scale objects. Representative research by Yang et al. [19]. demonstrates a 3D measurement system for precision shaft components utilizing line‐structured light projection integrated with deep learning (DL) methodologies.

Naturally, hybrid approaches integrating infrared light or structured light with multi‐camera systems for 3D modeling also exist because multi‐view stereo vision can significantly expand the measurement field and help resolve matching ambiguities. As demonstrated in the work of Wang et al. [20]. projected custom‐designed structured‐light dot patterns onto objects, proposing a single‐shot structured‐light method that leverages bi‐view geometry for 3D reconstruction; Gong et al. [21]. developed a quad‐view stereo vision measurement framework employing Gaussian Process (GP) regression with black‐and‐white stripe pattern illumination.

Stereo vision primarily relies on a dual‐camera system, operating on a principle analogous to human binocular vision. The average interpupillary distance in adults is approximately 65 mm [22]. When observing an object, the positional disparity between the two eyes results in distinct images being formed on each retina. These monocular images are subsequently transmitted via the optic nerves to the visual cortex, where neural processing integrates them into a 3D percept, a phenomenon defined as binocular stereopsis [15, 22, 23]. Similarly, in a stereo vision dual‐camera system, a fixed baseline distance exists between the two cameras. When the two cameras simultaneously capture the same object, a discernible disparity arises between the two images due to their distinct spatial positions. By analyzing and calculating this disparity, the depth information of the object can be derived [17], thereby enabling the reconstruction of objects in 3D space. This principle is exemplified in the work of Li et al. [24], who employed a GA‐DenseNet algorithm integrated with binocular stereo vision to achieve road pothole identification and 3D reconstruction.

In summary, despite enabling exceptional 3D detection and reconstruction capabilities, these 3D image acquisition solutions universally exhibit inherent limitations, including elevated equipment costs, computational complexity, and substantial power demands. The majority of commonly accessible images are captured using monocular cameras under unstructured illumination conditions. These images contain only 2D information. Reconstructing 3D representations from multiple 2D images of the same object captured from varied viewpoints necessitates multi‐view image fusion algorithms. These computational processes exhibit significant complexity and impose substantial computational burdens, consequently hindering rapid and efficient analysis.

Image recognition finds extensive application in colorimetric analysis, where contemporary mobile platforms such as smartphones enable efficient and accessible concentration detection through computational identification of concentration‐dependent color variations. Recent advances in DL have significantly enhanced the capabilities of colorimetric sensing systems, enabling rapid and accurate quantification of diverse analytes across environmental, industrial, and healthcare domains [25, 26, 27, 28, 29]. For instance, dual‐modal colorimetric/electrical systems enable ultrafast H_2_S detection [25], while recoverable hydrogel‐based colorimetric sensors offer reusable platforms for H_2_S monitoring [26], and flexible wearable patches allow real‐time ammonia leakage warning [27]. It has also empowered AI‐driven wearable sensors for gesture recognition [28] and multiplexed biomarker analysis in biofluids such as tears [29]. These studies highlight the potential of integrating artificial intelligence with colorimetric platforms for intelligent and portable sensing. Compared to paper‐based color sensors, gel sensors are widely used because they have a stronger absorption capacity for samples. The chromatic response of gel‐based sensors depends on both analyte concentration and volume [30]. Nevertheless, contemporary gel sensors predominantly disregard volumetric influences or require precise sample dosing [31]. While grid‐based calibration in single‐image acquisitions can partially normalize area variations induced by hydrogel swelling [32], it fails to accurately quantify volumetric changes along the vertical axis. This limitation fundamentally originates from the inherent inability of monocular imaging systems to acquire depth information perpendicular to the imaging plane.

In this work, our objective is to encode 3D information into images captured by a conventional monocular camera, using the acquired depth data to achieve precise calibration of colorimetric concentration. Unlike other multi‐camera systems, our strategy requires only a monocular camera and controlled illumination, significantly reducing cost and complexity. We employ a hydrogel carrier platform to develop a universally applicable shadow‐calibrated strategy. By introducing controlled illumination, our method establishes morphometric correlations between geometric features of cast shadows and 3D surface topography, enabling depth reconstruction from monocular images. This approach achieves comprehensive 3D depth information extraction within a simple 2D image. The proposed shadow‐assisted 3D reconstruction methodology significantly enhances detection accuracy by integrating CNN‐based analysis with volumetric shadow calibration, building upon conventional colorimetric frameworks. This synergistic approach establishes a novel paradigm for developing portable rapid‐sensing devices. Furthermore, an exploratory demonstration of its biomedical implementation in sweat analysis validates practical applicability for real‐life biospecimen characterization.

## 2 Experimental Section

### 2.1 Materials and Reagents

Acrylamide (AM) (AR, Macklin), Poly(vinyl alcohol) (PVA) Model 1799 (alcohol solubility 98%–99%, Macklin), N, N'‐methylene bisacrylamide (BIS) (AR, Macklin), Ammonium persulfate (APS) (AR, Macklin) and N,N,N',N'‐Tetramethylethylenediamine (TEMED) (99%, Macklin) are used. All materials and reagents are described in detail in the Supporting Information.

### 2.2 Preparation of the 3D Hydrogels

The fabrication procedure of 3D hydrogels is illustrated in Figure S1, utilizing polyvinyl alcohol (PVA, 0.15 g/L) and acrylamide (AM, 0.25 g/mL) as monomers, where PVA provides hygroscopic swelling properties to enhance absorption of indicator solutions and test analytes while AM imparts mechanical rigidity for improved structural support and easier demolding. The synthesis process begins with heating the optimized AM/PVA mixture (see Figure S2 for ratio) at 100°C under oil‐bath reflux with continuous stirring for 2 h. After cooling, 1 mL of the solution is transferred to a beaker and mixed sequentially with 50 µL of 0.1 g/mL ammonium persulfate (APS) as initiator, 50 µL of 0.01 g/mL N,N'‐methylenebisacrylamide (BIS) as crosslinker, and 10 µL of 50% N,N,N',N'‐tetramethylethylenediamine (TEMED) as accelerator, followed by thorough vortex mixing to ensure homogeneity. The precursor solution is then immediately injected into various molds (including cone, cube, cylinder, quadrangular pyramid and triangular pyramid shapes as shown in Figure S3) and allowed to polymerize at room temperature for 2 h. Finally, the 3D PAM/PVA hydrogel is obtained. The SEM images (Figure S4) reveal that the fabricated hydrogels possess a loose, porous microstructure with an average pore size of 19.93 ± 0.61 µm. This high surface area morphology enhances solution absorption and swelling capacity. From the FTIR spectroscopy analysis (Figure S5), it can be observed that the hydroxyl stretching vibration peak of PVA at 3282 cm^−^^1^, the N–H peak of the amide group in PAM at 3347 cm^−^^1^, and the C = O peak at 1657 cm^−^^1^ are all distinctly present in the PAM‐PVA spectrum, confirming the successful synthesis of the PAM‐PVA composite hydrogel.

### 2.3 Preparation of Indicator Solutions and 3D Colorimetric Patches

The indicator solutions are formulated as follows: (1) Zn^2+^ indicator. 0.002 g Zincon dissolved in 2 mL borate buffer (pH 9), 7 mL deionized water, and 1 mL ethanol, followed by sonication; (2) Ca^2+^ indicator. 0.007 g chlorophosphonazo mA mixed with 1 mL Na_2_HPO_4_ (0.1 M, pH 6.5) and 16 mL deionized water, then sonicated; (3) Glucose indicator. 0.0015 g 4‐AAP combined with 5 mL each of phenol (2 mg/mL in PBS), glucose oxidase (0.24 g/mL, 100 U/mg), and horseradish peroxidase (0.08 g/mL, 250 U/mg); (4) Lactate indicator. 0.01 g lactate oxidase (25 U/mg) and 0.001 g horseradish peroxidase (250 U/mg) dissolved in 10 mL water, then mixed 1:1 with 0.3 mg/mL TMB solution (prepared by dissolving 0.015 g TMB in pH 1 HCl and PBS dilution). For 3D hydrogel patch fabrication, the PVA/AM precursor solution (0.15 g/L PVA, 0.25 g/mL AM) is polymerized with APS/BIS/TEMED initiator system, molded into geometric shapes (cones, cubes, etc.), and immersed in respective indicator solutions for 3 h to obtain functionalized colorimetric sensors. The fully swollen and chromogenically developed 3D colorimetric patch is placed on grid paper, and the entire setup is transferred into a light‐proof chamber. Under controlled illumination at a specified angle, top‐view images are acquired using a digital camera to obtain the image dataset for colorimetric quantification.

## 3 Results and Discussion

Sweat, a non‐invasive source of clinically valuable biomarkers, offers significant diagnostic potential due to its facile collection and processing [33] and its composition's strong correlation with physiological status. Consequently, precise quantification of sweat biomarkers proves essential for applications spanning early disease detection, health monitoring, and sports medicine research.

**FIGURE 1:**
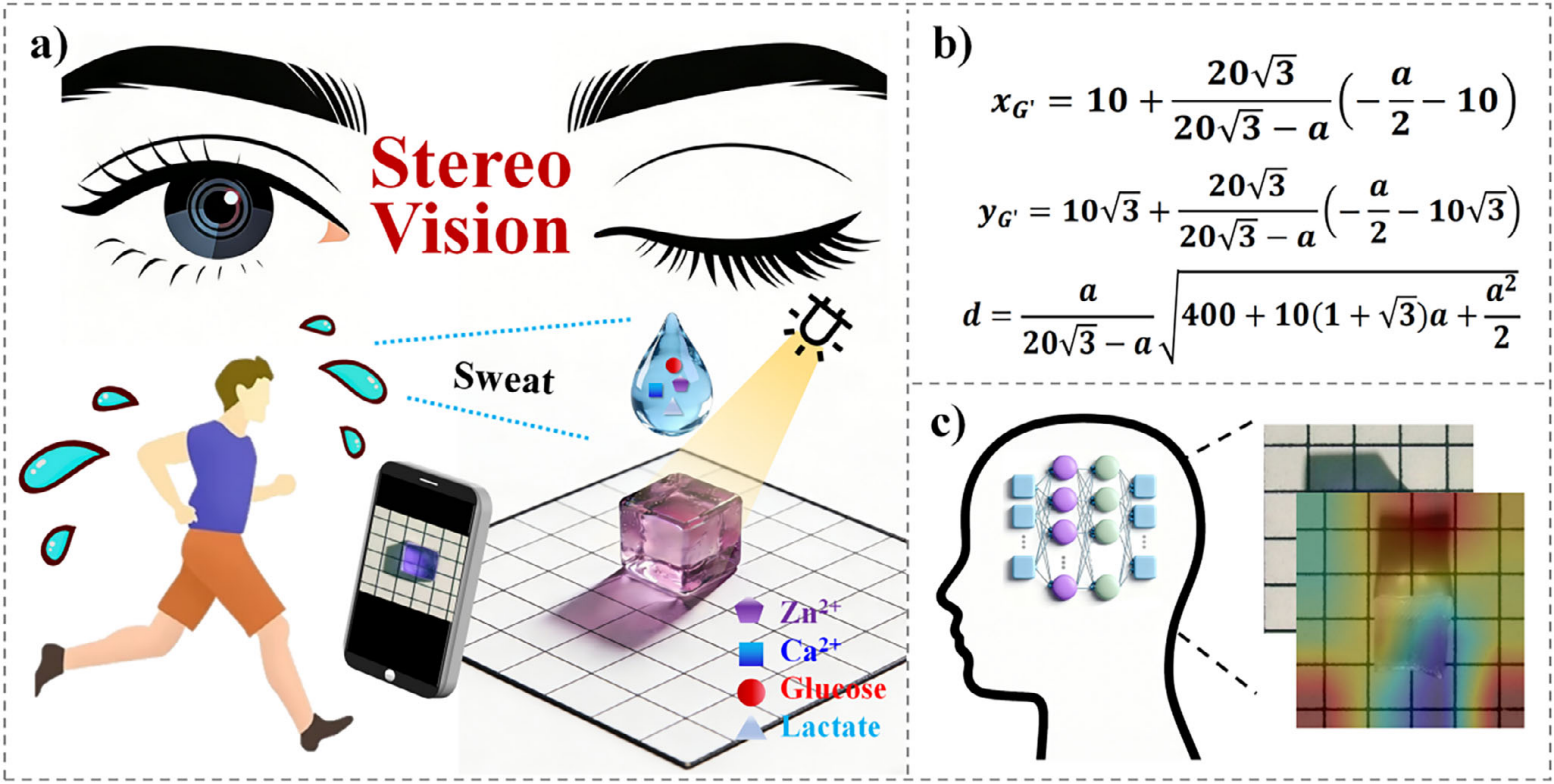
(a) Schematic illustration of the 3D shadow‐calibrated patch for sweat biomarker detection. (b) Mathematical principles of 3D shadow‐calibrated strategy and (c) visualization of the convolutional neural network's decision‐making mechanism.

This study introduces an explainable DL‐assisted, shadow‐calibrated 3D patch colorimetric sensing platform for sweat analysis (Figure 1a). The method involves depositing sweat samples into a cubic hydrogel patch. After allowing for complete swelling and full color development, the hydrogel patch is positioned on a grid platform. A controllable point light source is then innovatively positioned at a specific oblique angle. When illuminating the hydrogel cube, the light casts shadows onto the gridded base platform, enabling a top‐view camera to capture 2D images containing encoded 3D information through this shadow projection mechanism. Conventional stereo vision typically relies on binocular camera systems, mirroring human binocular perception. In this methodology, the imaging device functions as the “primary eye,” while the light source serves as an optical surrogate for the “secondary eye” (Figure 1a). This strategic design effectively addresses a fundamental limitation in conventional monocular top‐down imaging systems: their inherent inability to acquire depth‐resolved information along the axial swelling dimension of hydrogels. Such dimensional insensitivity would otherwise lead to chromatic distortions, ultimately degrading the precision of colorimetric measurements.

Theoretical derivation establishes a rigorous mathematical relationship (Figure 1b) between the geometric features of the projected shadow on the underlying grid plane (characteristic length *d*) and the 3D of the hydrogel (vertical edge length *a*). This foundational correspondence provides the theoretical basis for employing Convolutional Neural Networks (CNN) to analyze the image data (Figure 1c). By synergistically analyzing both chromatic information from color regions and depth information from shadow regions within the captured images via a CNN, this approach achieves precise volumetric calibration of hydrogel swelling. Consequently, it significantly enhances the accuracy of quantitative colorimetric analysis for sweat biomarker concentration.

### 3.1 Mathematical Principles of the Shadow‐Assisted Model and Explainable Deep Learning

**FIGURE 2:**
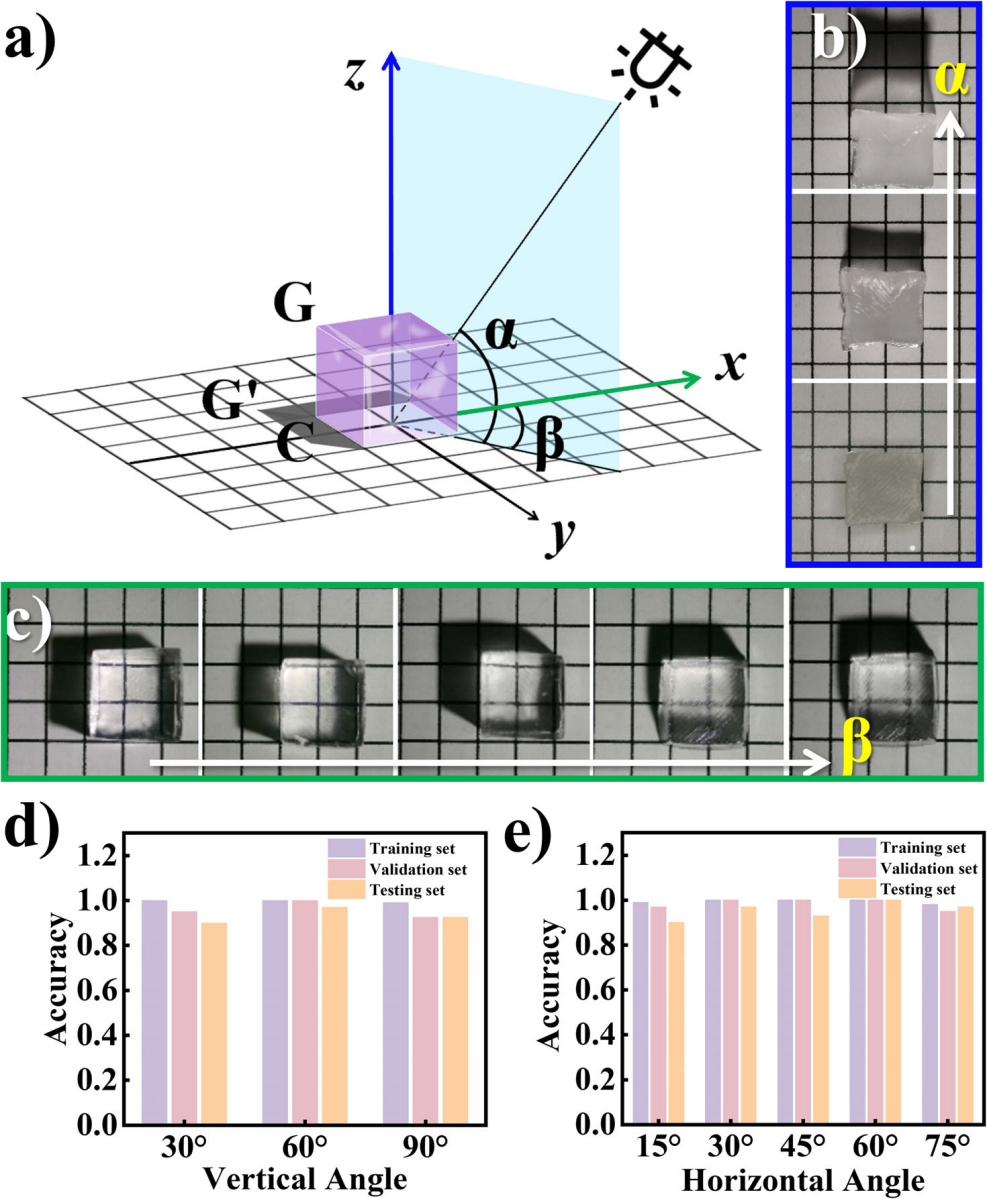
(a) Schematic of a fully swollen and chromatically developed 3D hydrogel specimen positioned on a gridded substrate. (b) Physical images of hydrogel specimens illuminated at elevation angles of 30°, 60°, and 90°. (c) Physical images of hydrogel specimens illuminated at azimuth angles of 15°, 30°, 45°, 60°, and 75°. (d) CNN classification accuracy corresponds to varied elevation angles. (e) CNN classification accuracy corresponds to varied azimuth angles.

Figure 2a illustrates the construction principle of the shadow projection model. A cubic hydrogel specimen with an assumed vertical edge length of *a*, pre‐loaded with a sweat sample and having undergone complete swelling and chromatic development, is positioned on a gridded substrate and enclosed within a light‐proof chamber. Imaging is performed vertically from above using a monocular camera. As monocular imaging inherently lacks depth information along the vertical axis of the 3D hydrogel, a point light source (S) is introduced such that its projection on the grid plane is positioned 20 cm from the center of the cubic hydrogel's base. This configuration projects depth information onto the 2D image plane through the resulting geometric shadow pattern. The elevation angle (*α*) and azimuth angle (*β*) of light source S are defined. To optimize model performance, illumination geometries are systematically evaluated using a CNN‐based classification framework. Cubic hydrogel specimens are first illuminated at elevation angles of 30°, 60°, and 90° (Figure 2b; Figure S6). Maximum classification accuracy of 97% is observed at *α* = 60° (Figure 2d; Figure S7), establishing this elevation as optimal for vertical illumination. Subsequently, with the elevation angle fixed at *α* = 60°, the azimuth angle *β* varies among 15°, 30°, 45°, 60°, and 75° (Figure 2c; Figure S8). Figure 2e demonstrates that an azimuth angle of *β* = 60° achieves perfect 100% classification accuracy (Figure 2e; Figure S9). Consequently, the illumination geometry with *α* = *β* = 60° is consistently employed for all subsequent image data acquisition.

**FIGURE 3:**
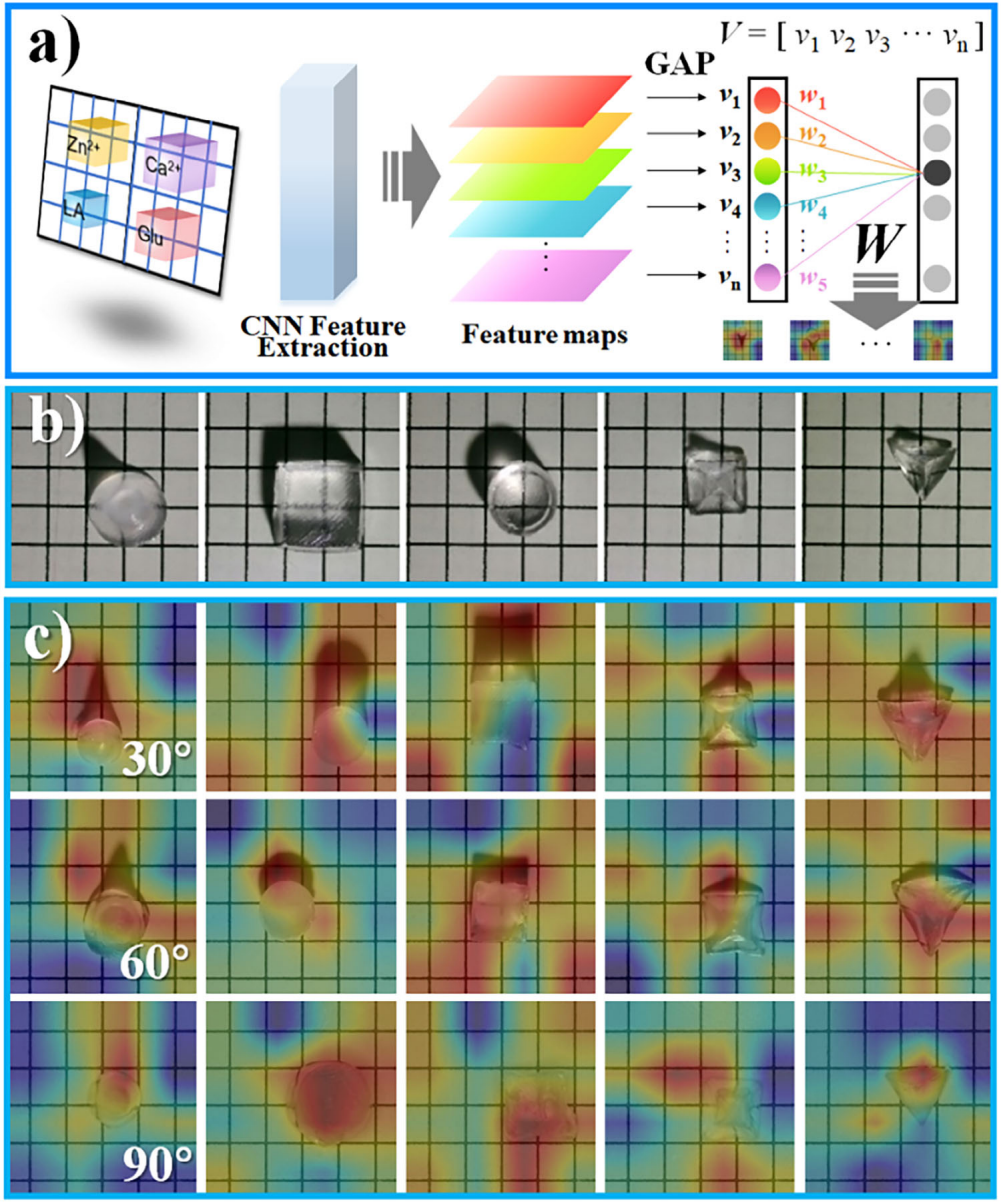
(a) Feature extraction based on the CAM mapping mechanism. (b) Physical images of five hydrogel patches with different shapes. (c) CAM heat maps generated by CNN for five hydrogel patches with different shapes under different light source angles.

DL models are frequently characterized as “black boxes” due to their opaque decision‐making processes and the inherent difficulty in interpreting internal decision‐making mechanisms [34]. To enhance model interpretability, we employed Class Activation Mapping (CAM) technology to visualize the CNN's decision process, thereby elucidating the foundation for its 100% classification accuracy. Figure 3a depicts the schematic diagram of the underlying decision mechanism in the CAM technique. We utilized this technique to characterize five hydrogels with distinct geometries under various illumination angles (Figure 3b). Within the heatmaps generated, regions exhibiting higher attention weights are represented in red, while areas of lower attention appear in blue (Figure 3c). Critical analysis of these visualizations reveals that the CNN not only autonomously identifies the spatial position and contour of the 3D hydrogel but also demonstrates pronounced focus on the geometric attributes of its projected shadow. This observation directly validates the substantive contribution of shadow‐derived features to the classification mechanism.

Furthermore, the theoretical soundness of the model design is substantiated through mathematical derivation. As illustrated in Figure 2a, a Cartesian coordinate system is established with its origin at the center of the cubic hydrogel specimen's base plane. Within this framework, the light source is denoted as point S $(10,10\sqrt{3},20\sqrt{3})$, a base vertex as point C $\left(-\frac{a}{2},-\frac{a}{2},0\right)$, and its corresponding vertex on the top surface as point G $\left(-\frac{a}{2},-\frac{a}{2},a\right)$. Given the rectilinear propagation of light, the intersection points of line SG with the *z* = 0 plane define the shadow point G’ corresponding to vertex G. The characteristic length of the projected shadow, CG’, serves as the quantifiable geometric feature for depth calibration.

The parametric equation of line SG is:

(1) $$\begin{array}{c} \left\{\begin{array}{c} x=10+t\left(-\frac{a}{2}-10\right)\\ y=10\sqrt{3}+t\left(-\frac{a}{2}-10\sqrt{3}\right)\\ z=20\sqrt{3}+t(a-20\sqrt{3}) \end{array}\right. \end{array}$$

Where *t* is the parameter, setting z = 0 reveals that,

(2) $$\begin{array}{c} t=\frac{20\sqrt{3}}{20\sqrt{3}-a} \end{array}$$

The coordinates of G’ are:

(3) $$\begin{array}{c} x_{G^{\prime }}=10+\frac{20\sqrt{3}}{20\sqrt{3}-a}\left(-\frac{a}{2}-10\right) \end{array}$$

(4) $$\begin{array}{c} y_{G^{\prime }}=10\sqrt{3}+\frac{20\sqrt{3}}{20\sqrt{3}-a}\left(-\frac{a}{2}-10\sqrt{3}\right) \end{array}$$

From the formula for the distance between two points, the distance *d* of CG’ can be expressed as:

(5) $$\begin{array}{c} d=\left|1-t\right|\sqrt{{\left(10+\frac{a}{2}\right)}^{2}+{\left(10\sqrt{3}+\frac{a}{2}\right)}^{2}} \end{array}$$

Substituting *t* and simplifying:

(6) $$\begin{array}{c} \left|1-t\right|=\frac{a}{20\sqrt{3}-a}\;\left(a<20\sqrt{3}\right) \end{array}$$

(7) $$\begin{array}{c} {\left(10+\frac{a}{2}\right)}^{2}+{\left(10\sqrt{3}+\frac{a}{2}\right)}^{2}=400+10\left(1+\sqrt{3}\right)a+\frac{a^{2}}{2} \end{array}$$

Finally, the distance *d* of CG’ be obtained as:

(8) $$\begin{array}{c} d=CG^{\prime }=\frac{a}{20\sqrt{3}-a}\sqrt{400+10\left(1+\sqrt{3}\right)a+\frac{a^{2}}{2}} \end{array}$$

From the final relation, it can be derived that a deterministic correspondence exists between the characteristic dimension of the shadow and the depth parameter a of the cubic hydrogel volume when the light source position is fixed. This correspondence effectively translates the depth information a, which cannot be measured directly, into the quantifiable 2D projection feature *d*, thereby establishing a physically interpretable theoretical foundation for single‐view 3D reconstruction. As the sample volume increased from 0 to 30 µL, the cubic volume increased from 22.69 to 44.57 mm^3^, and the characteristic length rose from 1.86 to 2.25 mm (Figure S10). These results indicate that an increase in sample volume directly leads to the enlargement of both the 3D hydrogel structure and its characteristic shadow length. Even though hydrogels of different geometries, such as cylinders, cones, triangular pyramids, and tetrahedral pyramids, exhibit differences in absorption capacity (Figure S11), their shadow features can all be effectively recognized by the CNN (Figure 3b,c), which demonstrates the broad applicability of the proposed volumetric hydrogel sensing method.

### 3.2 Colorimetric Sensing Mechanisms for Sweat Biomarkers

In recent years, the rapid advancements in wearable technology and personalized medicine have driven a growing demand for sweat analysis techniques [35, 36, 37, 38]. This necessitates detection methods that are not only accurate and reliable but also feature operational simplicity, cost‐effectiveness, and the capability for portable on‐site rapid testing. Colorimetric methods, which quantify biomarker concentrations by detecting chromogenic responses in samples, have gained widespread adoption due to their inherent visual detectability, procedural simplicity, and elimination of the need for sophisticated instrumentation.

**FIGURE 4:**
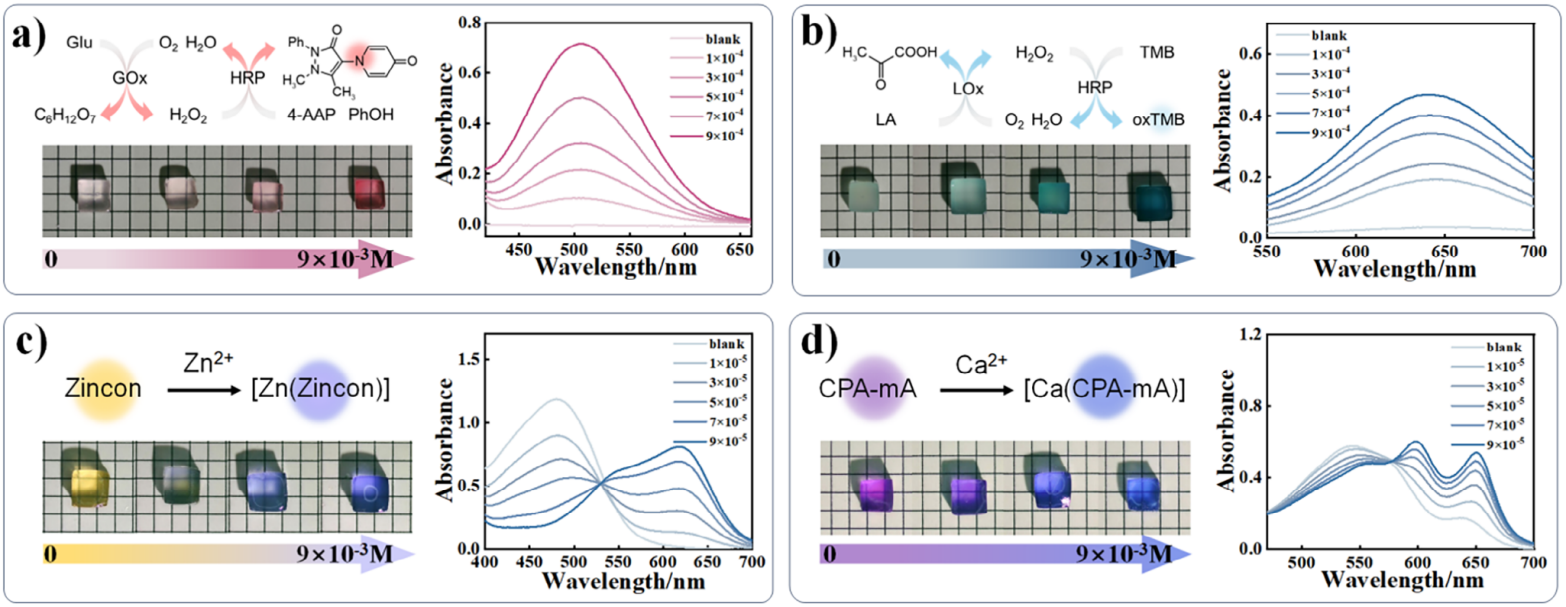
The colorimetric mechanisms and UV–vis absorption spectra for (a) glucose, (b) lactate, (c) zinc ions, and (d) calcium ions.

The composition of sweat serves as a vital indicator of human health. In this study, calcium ions, zinc ions, lactate, and glucose are selected as target analytes. A significantly elevated concentration of calcium ions in sweat is a diagnostic marker for cystic fibrosis [39], while also reflecting electrolyte imbalance and dehydration risk. Zinc, an essential trace element, plays a critical role in immune function, growth, development, and wound healing [40]. Its abnormal excretion may be associated with certain skin diseases, such as psoriasis and dermatophytosis [41]. Lactate concentration serves as a sensitive indicator for assessing exercise intensity and tissue hypoxia; it increases rapidly during strenuous physical activity, and an abnormal rise in critically ill patients may suggest metabolic crises such as shock or sepsis [42]. Glucose concentration correlates with blood sugar levels, and the detection of glucose in sweat represents an important approach for non‐invasive continuous monitoring of diabetes. The corresponding chemical colorimetric sensing mechanisms, UV spectral variations, and visual color changes for these four substances are illustrated below (Figure 4a–d).

Glucose oxidase (GOx), the sole natural enzyme capable of oxidizing glucose, catalyzes the oxidation of glucose to gluconic acid and H_2_O_2_ in the presence of oxygen. The generated H_2_O_2_ subsequently oxidizes 4‐aminoantipyrine (4‐AAP) and phenol under the catalysis of horseradish peroxidase (HRP), yielding a red‐colored quinone derivative (Figure 4a; Figure S12). This reaction corresponds to an increased absorption peak at 506 nm in the spectrum. Lactate detection employs lactate oxidase (LOx). In the presence of oxygen, LOx catalyzes the oxidation of lactate to pyruvate and H_2_O_2_. The resulting H_2_O_2_ subsequently oxidizes colorless 3,3’,5,5’‐tetramethylbenzidine (TMB) into blue oxTMB (Figure 4b; Figure S13), corresponding to an increased absorption peak at 640 nm in the spectrum. For zinc ion detection, the mechanism relies on a colorimetric transition from yellow to purple induced by Zincon (Figure 4c; Figure S14). The corresponding spectral changes exhibit a decreased absorption peak at 479 nm and an increased peak at 617 nm. Calcium ion quantification is achieved through a chromogenic reaction of chlorophosphonazo mA (CPA‐mA), which transitions from purple to blue (Figure 4d; Figure S15). The associated spectral profile shows reduced absorption at 543 nm and enhanced peaks at 598 nm and 649 nm. Each 3D colorimetric hydrogel patch exhibits good selectivity with no obvious cross‐interference (Figure S16).

### 3.3 DL‐Assisted Sweat Sensing and Actual Sweat Sample Analysis

Based on the aforementioned colorimetric sensing mechanisms, we collected 9000 image datasets using a 3D “shadow‐calibrated” method. The concentration range for all four substances is 1 × 10^−5^ to 9 × 10^−3^ mol·L^−1^, with each concentration being applied to the 3D hydrogel in volumes of 10–30 µL. The entire dataset of each substance is randomly divided into three subsets: training set, validation set, and test set, at a ratio of 8:1:1. The training set is utilized for model training, the validation set for hyperparameter tuning and preliminary model evaluation, and the test set for assessing the final model's generalization ability. To verify the accuracy of the established model, we employ various quantitative performance metrics for evaluation, including the coefficient of determination (R^2^), mean squared error (MSE), root mean squared error (RMSE), and mean absolute error (MAE).

**FIGURE 5:**
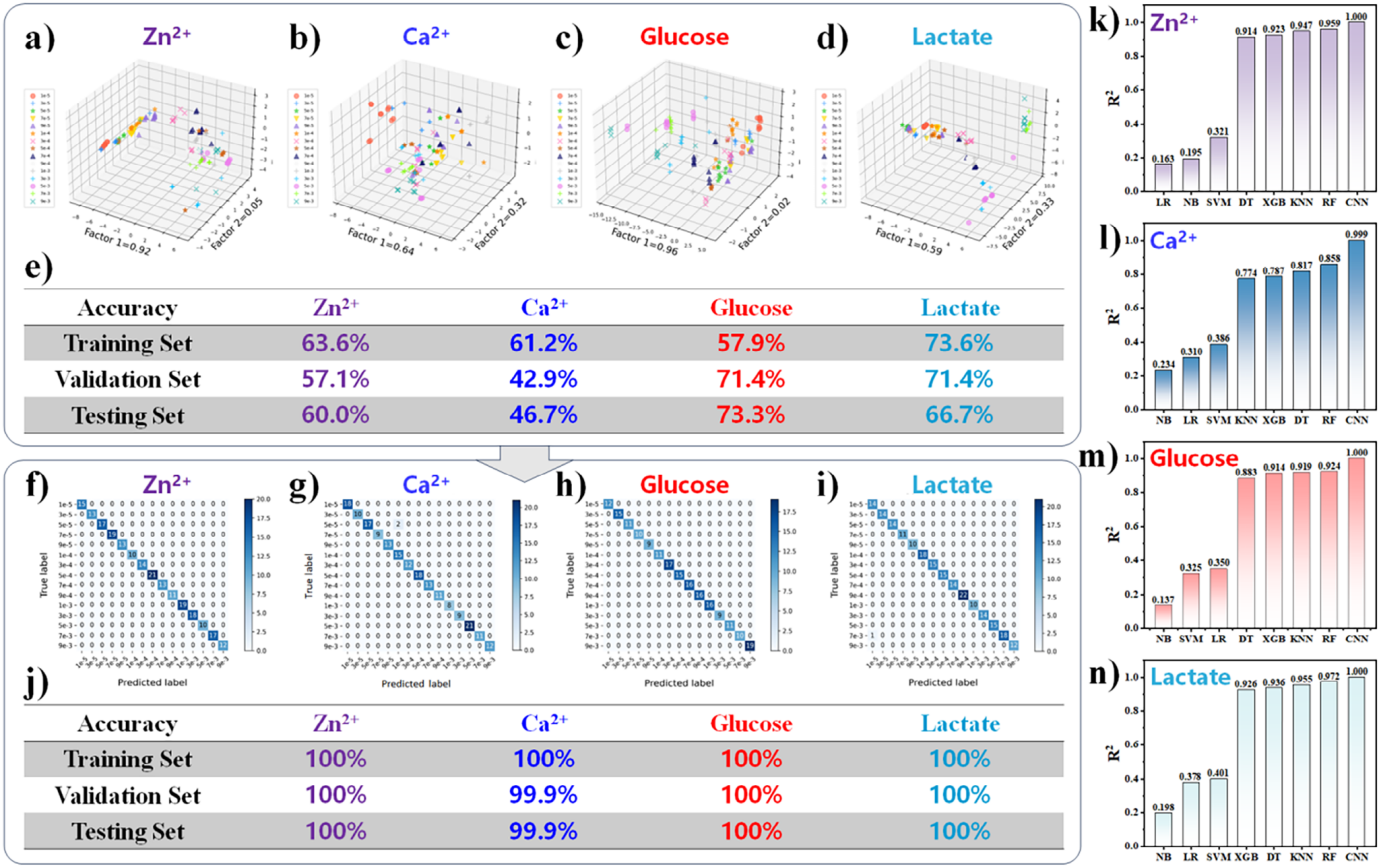
The 3D LDA score plot for (a) Zn^2+^, (b) Ca^2+^, (c) glucose, and (d) lactate. (e) Classification accuracy for Zn^2+^, Ca^2+^, glucose, and lactate on the training set (80% data), validation set (10% data), and testing set (10% data) based on LDA models. (f–i) Confusion matrixes of CNN prediction for Zn^2+^, Ca^2+^, glucose, and lactate. (j) Classification accuracy for Zn^2+^, Ca^2+^, glucose, and lactate on the training set (80% data), validation set (10% data), and testing set (10% data) based on CNN models. (k–n) Comparison of prediction accuracies for the sweat biomarkers by employing different classification models, including DT, KNN, LR, NB, RF, SVM, XGBoost, and CNN on the training set, validation set, and testing set.

Initially, we employ a Linear Discriminant Analysis (LDA) model to classify concentrations of the four sweat biomarkers (Figure 5a–e). Disappointingly, the LDA model failed to completely distinguish all sweat biomarkers, with significant overlapping observed among clusters. This indicates that the LDA model cannot meet the requirements for multivariate and high‐dimensional sensitive colorimetric analysis.

Then, we employ seven machine learning (ML) methods: Decision Tree (DT), Random Forest (RF), K‐Nearest Neighbors (KNN), Logistic Regression (LR), Naive Bayes (NB), Support Vector Machine (SVM), and XGBoost (XGB), to classify the four sweat biomarkers, with the results and detailed parameters provided in Figure 5k–n and Figures S17–S23 and Table S1, respectively. In an effort to bypass the laborious process of manually extracting and calculating RGB feature values, we also utilize a CNN to process the colorimetric images and construct a non‐linear model (Figure 5f–j; Figures S24–S27), with the parameters of the employed CNN classification algorithm detailed in Table S2. The results reveal a clear stratification in the performance of traditional machine learning models across the four tasks. Tree‐based models (RF, XGBoost, DT) exhibit excellent performance, which can be attributed to their inherent ability to capture nonlinear relationships between features and targets, while effectively enhancing generalization through ensemble learning strategies. KNN also demonstrates strong performance, primarily because colorimetric data tend to form clusters of similar samples in feature space, and KNN's distance‐based classification mechanism naturally leverages this local structure. In contrast, linear models such as LR perform poorly, fundamentally due to the nonlinear relationship between analyte concentration and color response, which renders linear decision boundaries inadequate for partitioning the feature space regions corresponding to different concentrations. The suboptimal performance of SVM may be related to its sensitivity to parameter selection and the difficulty of optimization in high‐dimensional, large‐sample settings. Naive Bayes exhibits the poorest performance, stemming from its assumptions of feature independence and specific distribution patterns, which lead to distorted probability estimates.

Compared to the aforementioned traditional machine learning methods, CNN demonstrates superior performance due to its inherent advantages in multimodal feature fusion (Figure 5j). CNN not only circumvents the subjectivity and complexity inherent in manual feature design and extraction, but also captures the intrinsic correlation between color variation and volumetric swelling, thereby significantly enhancing the accuracy of concentration inversion. In contrast, traditional machine learning models struggle to effectively utilize multimodal information and fail to fit complex nonlinear relationships, making it difficult for them to achieve comparable performance levels.

**FIGURE 6:**
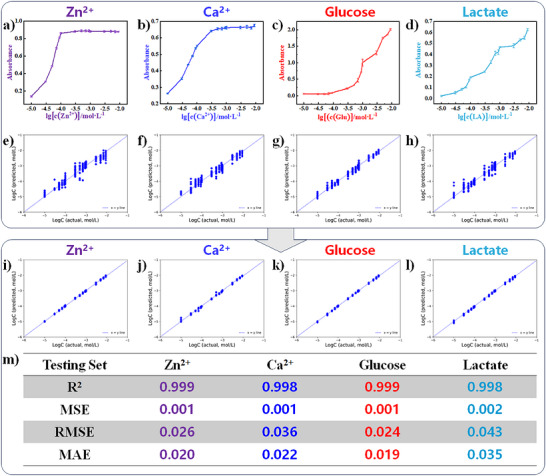
(a) Optical images and absorption spectrum (617 nm) dependence on Zn^2+^; (b) Optical images and absorption spectrum (649 nm) dependence on Ca^2+^; (c) Optical images and absorption spectrum (506 nm) dependence on glucose, and (d) optical images and absorption spectrum (640 nm) dependence on Lactate. (e–h) The concentration matching of actual measurement values and CNN predicted values uncalibrated processing. (i–l) The concentration matching of actual measurement values and CNN predicted values by shadow‐calibrated processing. (m) Regression evaluation metrics (MAE, RMSE, R^2^) for CNN concentration prediction on the test set.

Figure 6a–d shows the corresponding response spectra of selected sweat biomarkers at different gradient concentrations. We perform the analysis using a CNN quantification model, with its detailed architecture provided in Table S3. Figure 6e–h presents the CNN nonlinear regression results without “shadow‐calibrated”, demonstrating performance with test‐set R^2^ values ranging from 0.931 to 0.983. However, implementing the “shadow‐calibrated” imaging technique significantly improved the correlation coefficients to R^2^>0.998 (Figure 6i–m). Moreover, for Zn^2+^, Ca^2+^, glucose, and lactate, the MSE reductions reach 98.8%, 96.2%, 95.6%, and 94.8%, respectively; the RMSE reductions are 88.9%, 80.5%, 79.0%, and 77.2%; and the MAE reductions are 88.9%, 85.2%, 77.2%, and 73.6% (Figure S28). This performance significantly surpasses that of the CNN without the “shadow‐calibrated” strategy, as well as the results from traditional machine learning methods (Figures S28 and S29 and Tables S4–S9). This intuitive and substantial performance improvement strongly demonstrates the indispensability and advantages of the shadow‐calibrated strategy.

**FIGURE 7:**
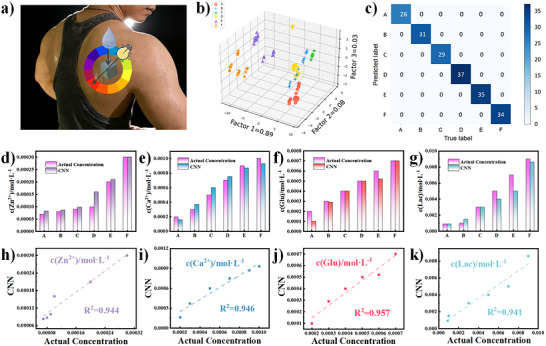
(a) Sweating. Classification results of six artificial sweat samples based on the (b) LDA model and (c) CNN model. (d–g) Comparison between the regression results of six artificial sweat samples using the CNN algorithm based on the shadow‐calibrated 3D hydrogel patch and the actual values. (h–k) The correlation analysis of actual measurement values and CNN‐predicted values.

The concentrations of representative components in human sweat (Figure 7a) under physiological conditions fall within the following ranges: calcium ions approximately 1 × 10^−4^ to 10^−3^ mol·L^−1^, zinc ions 1 × 10^−6^ to 10^−4^ mol·L^−1^, glucose 1 × 10^−5^ to 10^−4^ mol·L^−1^, and lactate 1 × 10^−3^ to 10^−2^ mol·L^−1^ [43, 44]. We prepared six artificial sweat samples within the normal human physiological concentration range for analysis. The LDA model (Figure 7b; Figure S30) could not accurately distinguish individual differences, whereas the CNN model (Figure 7c; Figure S31) achieves perfect matching. The CNN regression model demonstrates excellent predictive accuracy for these artificial sweat samples (Figure 7d–g), showing strong agreement with the actual concentrations (Figure 7h–k). The high determination coefficient can be attributed to the unique advantages of the shadow‐calibrated sweat sensing methodology. To further investigate the underlying mechanism, we employ CAM to visualize the attention regions of the CNN during the regression task (Figure S32). The results reveal that CNN not only focuses on the chromogenic regions within the 3D hydrogel patch images, but also attends to the positional, dimensional, and geometric characteristics of the accompanying shadows. This observation establishes a direct link between the CNN's decision‐making process and the sensing mechanism underlying the shadow‐calibrated strategy. Specifically, the attention on chromogenic regions corresponds to the fundamental principle of conventional colorimetric analysis, wherein the analyte concentration dictates the extent of color change. Meanwhile, the attention on shadow regions reflects the network's ability to infer 3D morphological information, namely the degree of volumetric change induced by hydrogel swelling, from 2D images. This dual‐attention mechanism aligns precisely with the core principle of our approach: accurate colorimetric quantification requires simultaneous consideration of both color intensity and the swelling volume of the hydrogel carrier. Consequently, the CAM heatmaps provide critical insights into the CNN's decision‐making process regarding the sensor's chemical information, further validating the reliability and scientific merit of the “chromaticity‐volume” co‐analysis strategy for colorimetric sensing.

## 4 Conclusions

In summary, we propose a “shadow‐calibrated” strategy that reconstructs 3D morphology by analyzing geometric features of object shadows under controlled illumination. Combined with an explainable DL model, this work achieves high‐precision detection of sweat biomarkers. And we have established the quantitative relationship between cubic hydrogel swelling depth *a* and shadow characteristic length *d*. This foundational correspondence provides the theoretical basis for employing CNN to analyze the image data. The CNN model trained on about 9000 shadow‐calibrated images achieves classification accuracies of 99%–100% for Zn^2+^, Ca^2+^, glucose, and lactate. In regression analysis, the shadow‐calibrated DL approach significantly improves the test set R^2^ from a range of 0.931–0.983 (uncalibrated) to above 0.998, with reductions of at least 94.8%, 77.2%, and 73.6% in MSE, RMSE, and MAE, respectively. These results conclusively demonstrate the pivotal role of shadow‐based 3D calibration in enhancing quantitative precision. Within physiological sweat concentration ranges, the CNN model achieves exceptional agreement between predicted and true values in artificial sweat samples, with R^2^ of 0.941–0.957, demonstrating robust performance for practical detection requirements. CAM visualizations reveal that the CNN's decision‐making process simultaneously incorporates both chromogenic response regions and shadow geometric features, significantly enhancing model interpretability. This strategy demonstrates compatibility with diverse regular hydrogel geometries and biomarkers, requiring only a monocular camera and controlled illumination. It thereby establishes a paradigm for developing highly accurate, low‐cost, and portable point‐of‐care testing (POCT) devices.

## Funding

National Natural Science Foundation of China (22474049), Guangdong Provincial Key Laboratory of Speed Capability Research (Grant No. 2023B1212010009), and the Key Research and Development Project of Guangdong Province (2023A1515011031).

## Conflicts of Interest

The authors declare no conflicts of interest

## Supporting information




**Supporting File**: advs75171‐sup‐0001‐SuppMat.docx.

## Data Availability

The data that support the findings of this study are available from the corresponding author upon reasonable request.
