# Supplementary material for: Shadow‐Calibrated Stereo Vision for Colorimetric Sweat Analysis
Source: Adv Sci (Weinh). 2026 Apr 7;13(36):e75171. doi: 10.1002/advs.75171 (PMC13317592; doi:10.1002/advs.75171)
Supplement: Supplementary file 1 — Supporting File: advs75171‐sup‐0001‐SuppMat.docx. [file ADVS-13-e75171-s001.docx]

Supporting Information

Shadow-Calibrated Stereo Vision for Colorimetric Sweat Analysis

Ting Xiao, Yuwen Yan, Miaorong Lin, Jiahui Chen, Jianxin Meng, Xiang Cui*, Peng Zhang* and Fengyu Li*

Ting Xiao, Yuwen Yan, Miaorong Lin, Jiahui Chen, Jianxin Meng, Peng Zhang, Fengyu Li

College of Chemistry and Materials Science, Guangdong Provincial Key Laboratory of Speed Capability Research, Su Bingtian Center for Speed Research and Training, Jinan University, Guangzhou 510632, China.
E-mail: [lifengyu@jnu.edu.cn](mailto:lifengyu@jnu.edu.cn) (Fengyu Li) & 24571565@qq.com (Peng Zhang)

Xiang Cui

Department of Orthopedics, Chinese PLA General Hospital, National Clinical Research Center for Orthopedics, Sports Medicine & Rehabilitation, Beijing 100853, China.

E-mail: cuixiang0828@163.com

Fengyu Li
College of Chemistry, Zhengzhou University, Zhengzhou 450001, China.

1. ***Materials and Reagents***

All chemical reagents are obtained commercially without further purification, including Acrylamide (AM, 99%), Poly(vinyl alcohol) (PVA, Model 1799), N, n-methylene bisacrylamide (BIS, 99%), Ammonium persulfate (APS, 99.99%), N,N,N',N'-Tetramethylethylenediamine (TEMED, 99%), Zincon indicator, CPA-mA indicator, Calcium chloride (CaCl_2_), Lactate Oxidase (LOx, 25U/mg), glucose oxidase (GOx, 100 U/mg), 4-Aminoantipyrene (4-AAP, 98%), Phenol (AR) from Shanghai Macklin Bio-Chem Technology Co., Ltd, Lactate (85%) from Shanghai Dingmiao Chemistry Co., Ltd., Horseradish peroxidase (HRP, 250 U/mg) from Shanghai Yuanye Bio-Technology Co., Ltd, 3,3',5,5'-Tetramethylbenzidine (TMB, 99.94%) from Bide Pharmatech Ltd., Phosphate buffered saline (PBS, 0.1 mol/L) from Shanghai Aladdin Bio-chemical Technology Co., Ltd., Zinc chloride (ZnCl_2_) from Tianjin Damao Chemical Reagent Partnership Enterprise. These reagents together provide the necessary chemical environment for the experiment.

1. ***Preparation Method of PAM-PVA Hydrogel***

The fabrication process for three-dimensional (3D) hydrogels is outlined in **Figure S1**, employing polyvinyl alcohol (PVA, 0.15 g/L) and acrylamide (AM, 0.25 g/mL) as monomers. PVA imparts hygroscopic swelling properties, which enhance the absorption of indicator solutions and test analytes, while AM contributes mechanical rigidity, improving structural support and facilitating easier demolding. The synthesis begins by heating an optimized mixture of AM and PVA (with the specific ratio detailed in Figure S2) at 100°C under oil-bath reflux with continuous stirring for 2 hours. After cooling, 1 mL of this solution is transferred to a beaker and sequentially combined with 50 μL of 0.1 g/mL ammonium persulfate (APS) as an initiator, 50 μL of 0.01 g/mL N,N'-methylenebisacrylamide (BIS) as a crosslinker, and 10 μL of 50% N,N,N',N'-tetramethylethylenediamine (TEMED) as an accelerator. The mixture is then thoroughly vortexed to ensure homogeneity. Immediately after, the precursor solution is injected into various molds—including cone, cube, cylinder, quadrangular pyramid, and triangular pyramid shapes, as illustrated in **Figure S3**, and allowed to polymerize at room temperature for 2 hours, resulting in the formation of the 3D PAM/PVA hydrogel.

To assess swelling performance, hydrogels prepared with different AM/PVA ratios (as shown in **Figure S2**) are first weighed to determine their initial mass (W_0_). They are subsequently immersed in water for 2 hours and reweighed to obtain the swollen mass (W_1_). The swelling ratio is calculated using the formula:

**Swelling ratio (%) = [(W_1_- W_0_) / W_0_] × 100%**

Following optimization, it is found that the hydrogel derived from a precursor solution containing 3 mL of 0.25 g/L PVA, 1 g of AM, and 2 mL of deionized water demonstrates the highest swelling performance.


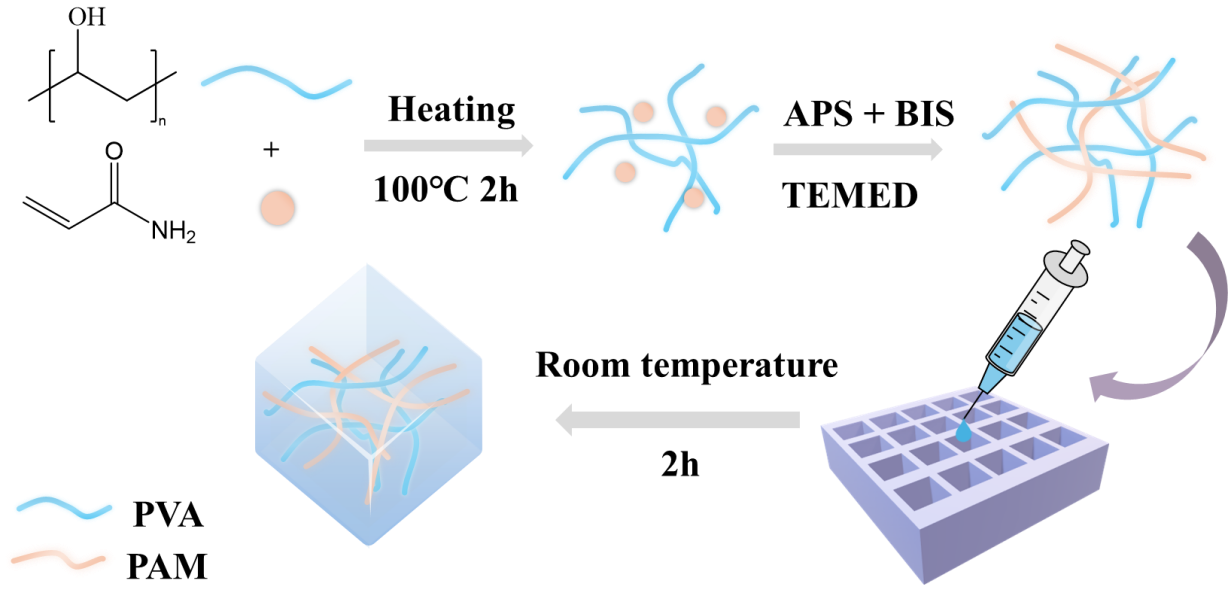


**Figure S1.** Preparation procedure of the PAM-PVA hydrogel cube.


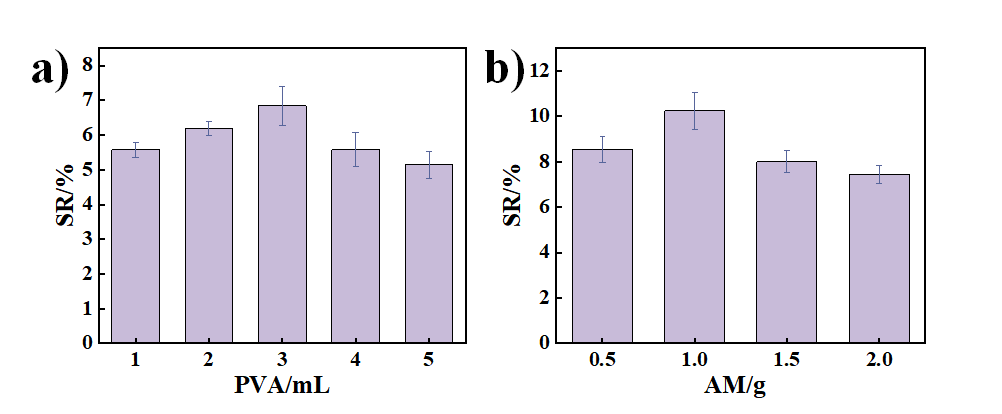


**Figure S2.** Optimization of a) PVA and b) AM ratios in the 3D hydrogel.


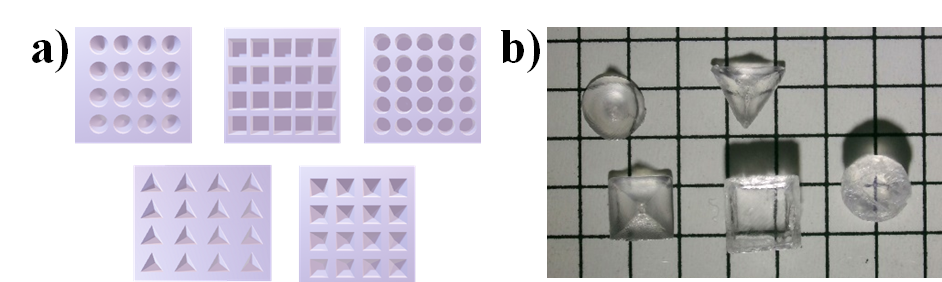


**Figure S3.** a) Molds with conical, cylindrical, cubic, square-pyramidal, and triangular-pyramidal shapes, and b) photographs of the corresponding 3D hydrogels after demolding.

1. ***Characterization of the PAM-PVA 3D Hydrogel.***


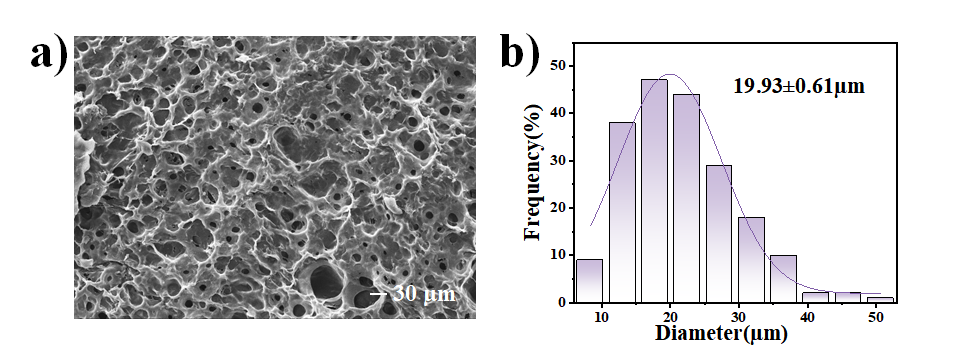


**Figure S4.** a) SEM image of PAM-PVA hydrogel and b) corresponding pore size distribution.

**Figure S5.** The FTIR spectrum of the PAM-PVA hydrogel.

1. ***Optimization of Light Source Angle.***

The 3D hydrogels with different geometries are initially illuminated at vertical angles of 30°, 60°, and 90° (**Figure S6**). The highest classification accuracy of 97% is achieved at a vertical illumination angle (α) of 60° (**Figure 2d**). Subsequently, while maintaining the optimal vertical angle (α = 60°), we vary the horizontal illumination angle (β) through 15°, 30°, 45°, 60°, and 75° (**Figure S8**). As demonstrated in **Figure 2e**, a perfect classification accuracy of 100% is attained when the horizontal angle (β) is also set to 60°. Therefore, the optimal illumination configuration is determined to be α = β = 60°.


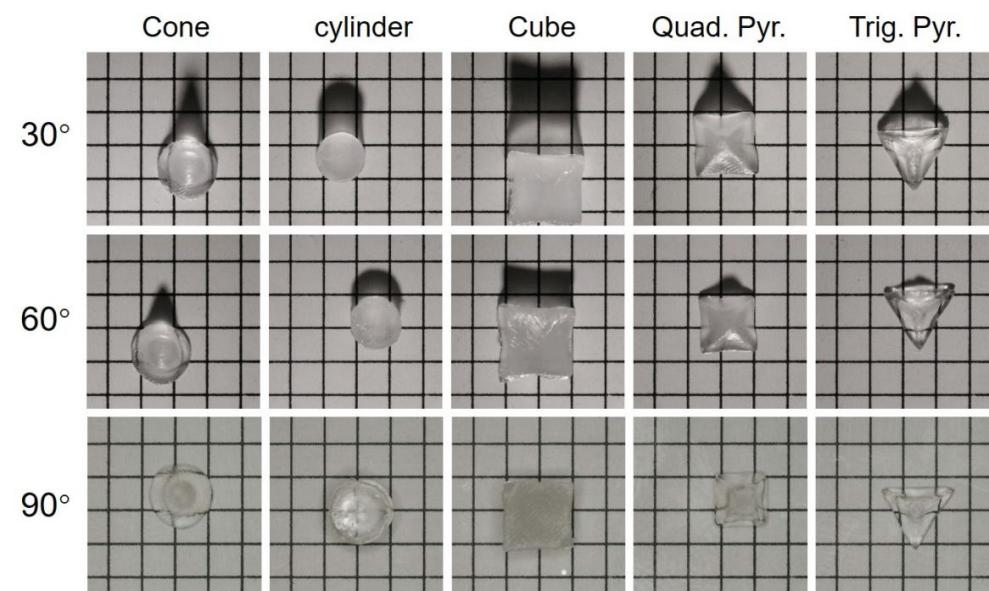


**Figure S6.** Photographs of 3D hydrogels with different geometries under varying vertical illumination angles. Abbreviation: Trig. Pyr. (trigonal pyramid), Quad. Pyr. (quadranglar pyamid).


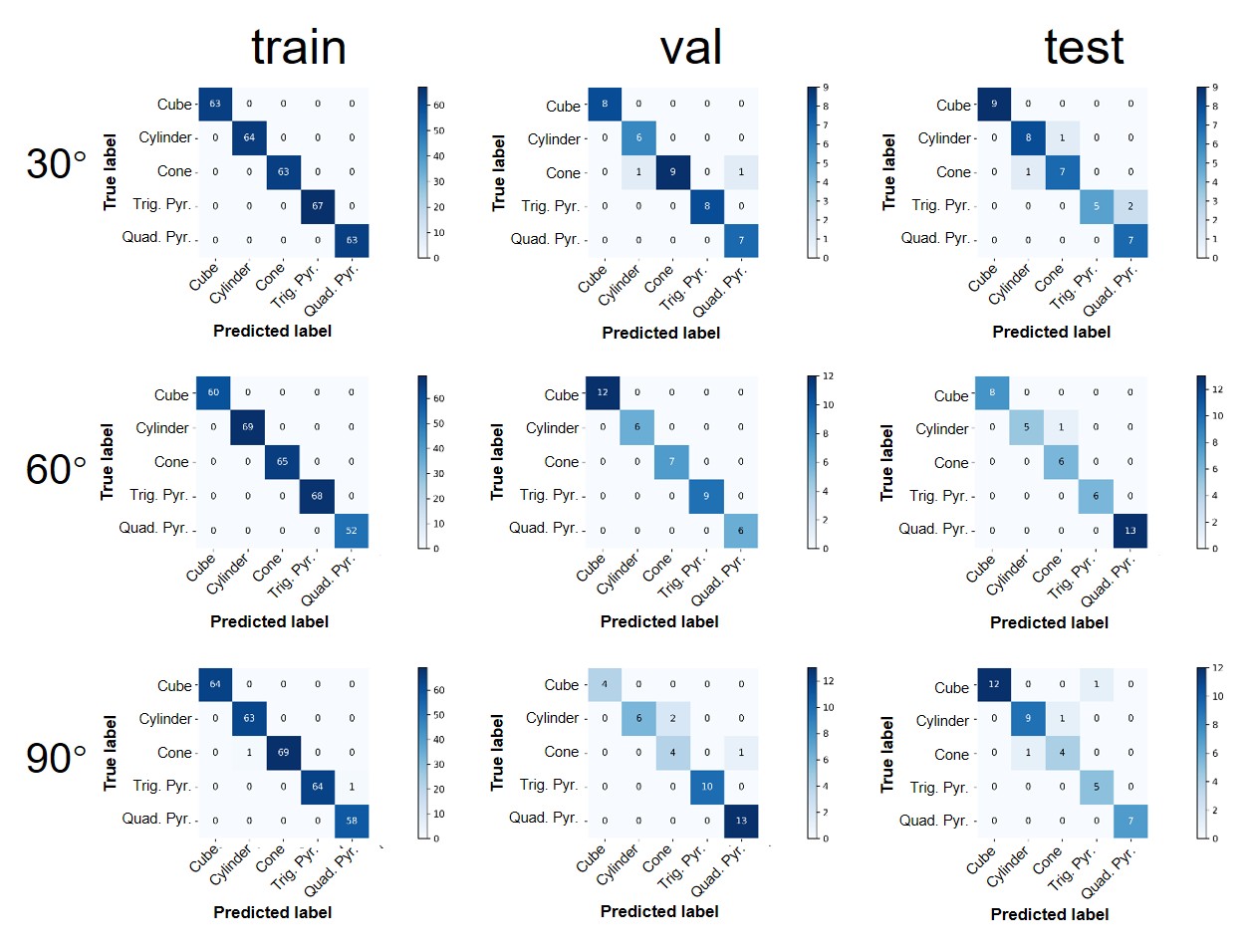


**Figure S7.** Confusion matrices for CNN-based classification of 3D hydrogels with distinct geometries under varying vertical illumination angles. Abbreviation: Trig. Pyr. (trigonal pyramid), Quad. Pyr. (quadranglar pyamid).


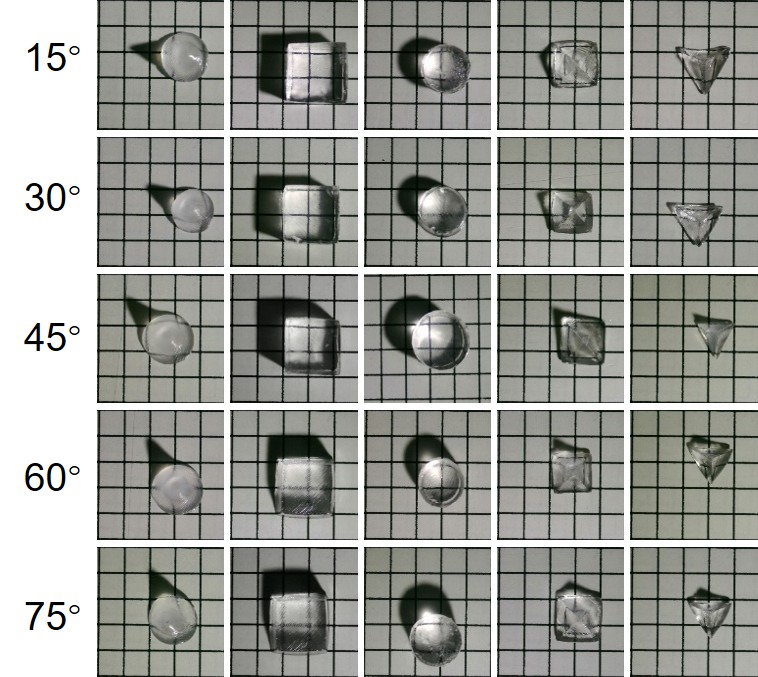


**Figure S8.** Photographs of 3D hydrogels with distinct geometries under varying horizontal illumination angles at a fixed vertical angle of 60°.


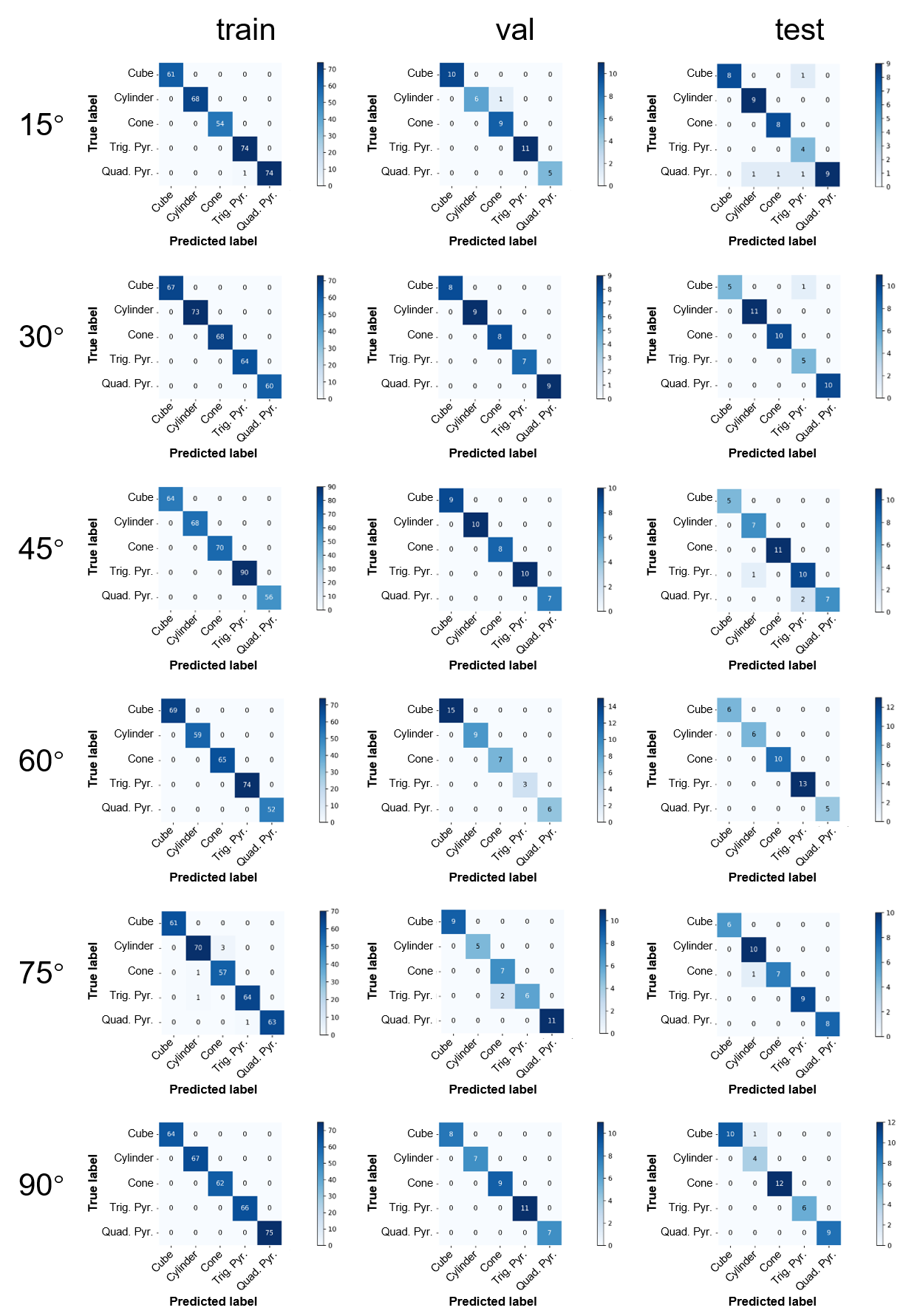


**Figure S9.** Confusion matrices for CNN-based classification of 3D hydrogels with distinct geometries under varying horizontal illumination angles at a fixed vertical angle. Abbreviation: Trig. Pyr.(trigonal pyramid), Quad. Pyr.(quadranglar pyamid).

1. ***The effect of the added sample volume on the 3D structure of the cubic hydrogel.***


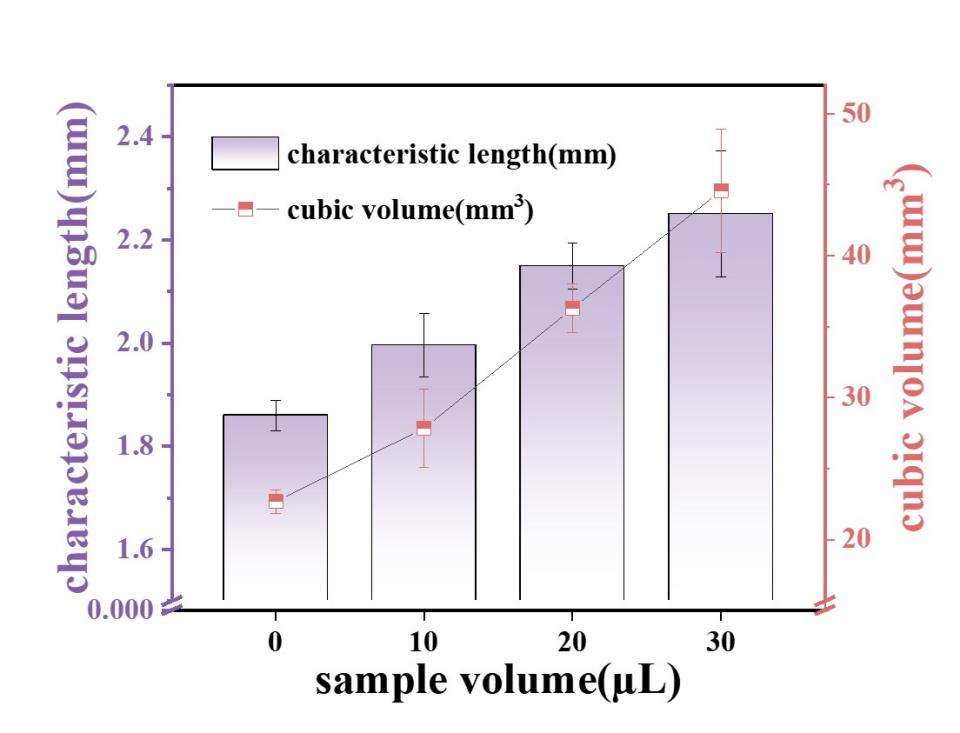


**Figure S10.** Variation of 3D cubic hydrogel volume and characteristic shadow length with sample volume.


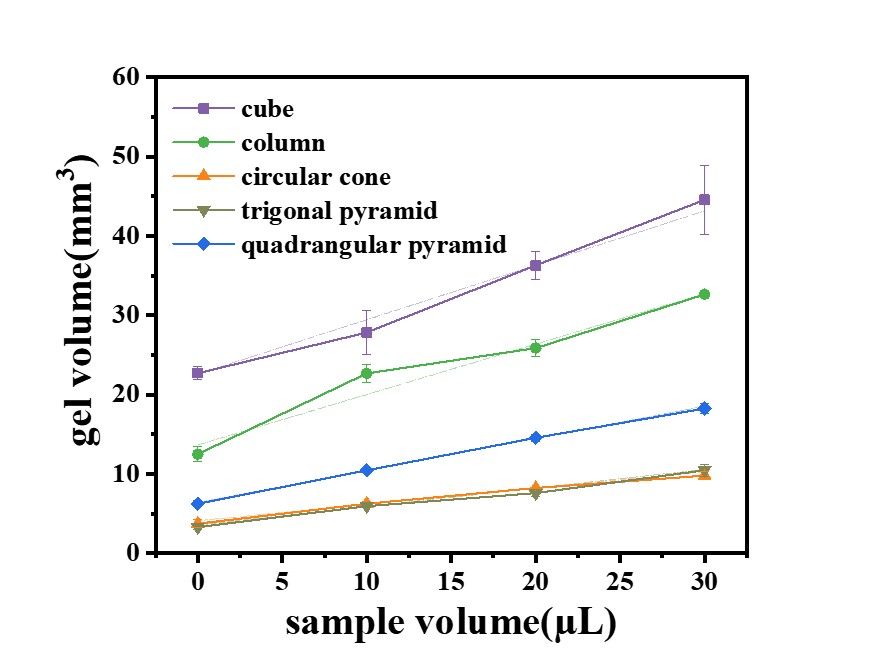


**Figure S11.** The effect of the added sample volume in hydrogels of different geometries on the volume of the cubic hydrogel.

1. ***Chromogenic Reaction in Indicator-Encapsulated 3D Hydrogels.***


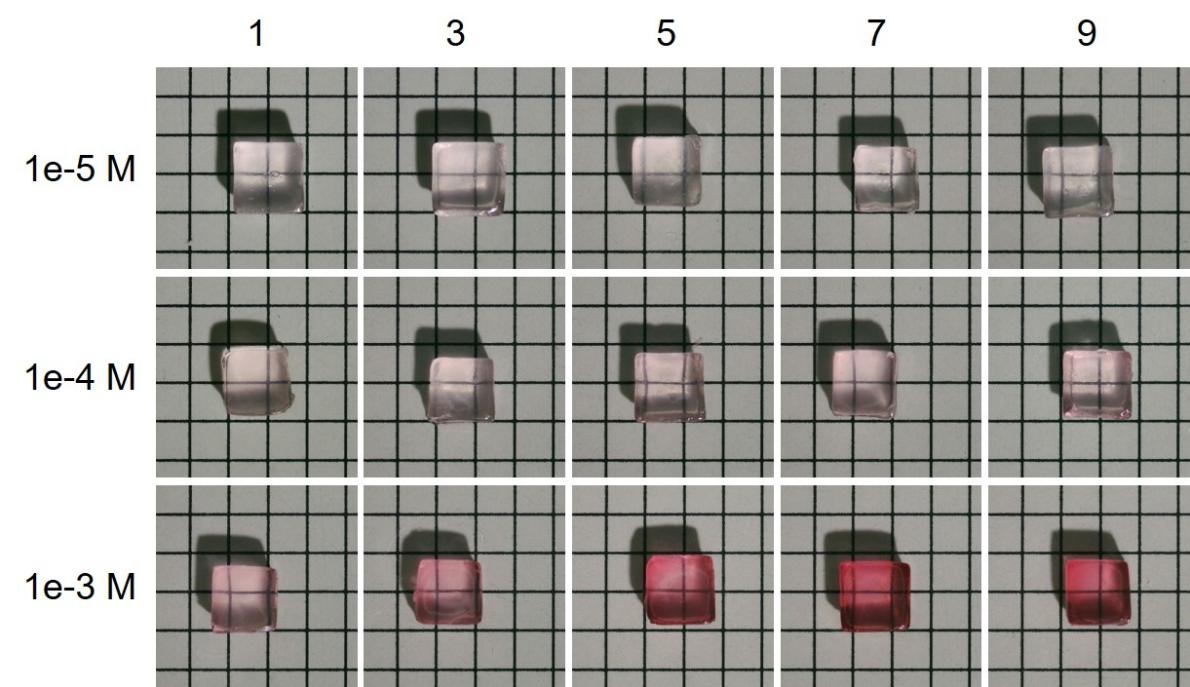


**Figure S12.** Photographic demonstration of colorimetric responses in glucose-indicator-encapsulated 3D hydrogels to varying glucose concentrations.


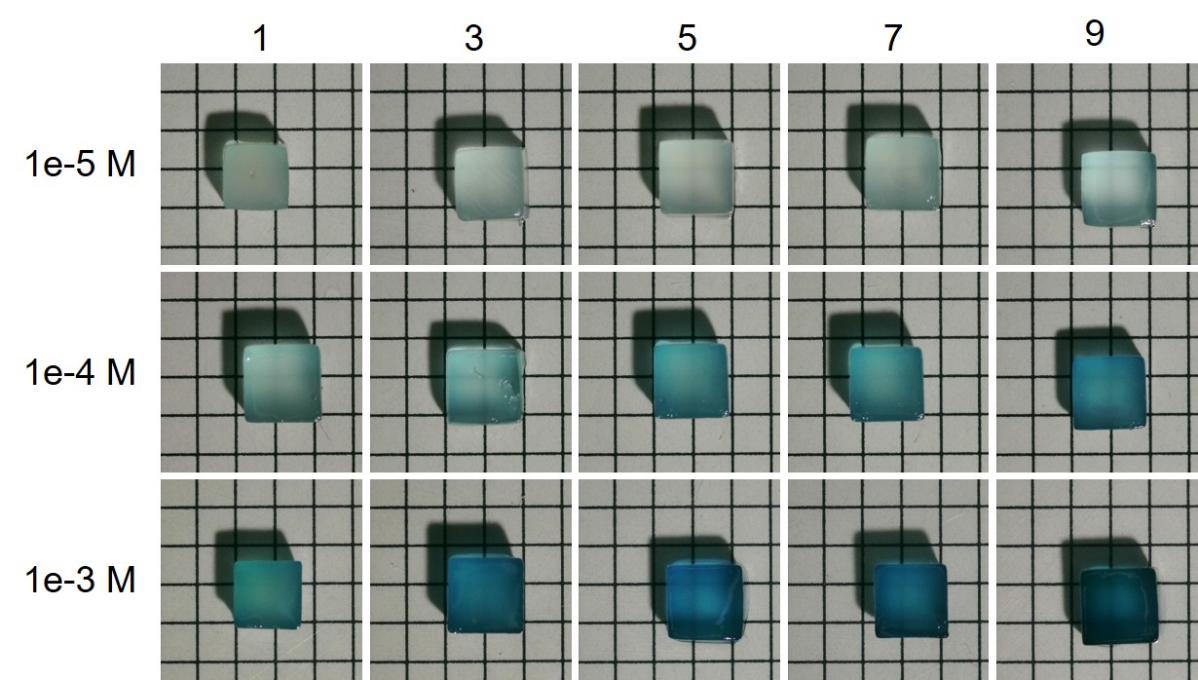


**Figure S13.** Colorimetric response of lactate-indicator-encapsulated 3D hydrogels to varying lactate concentrations.


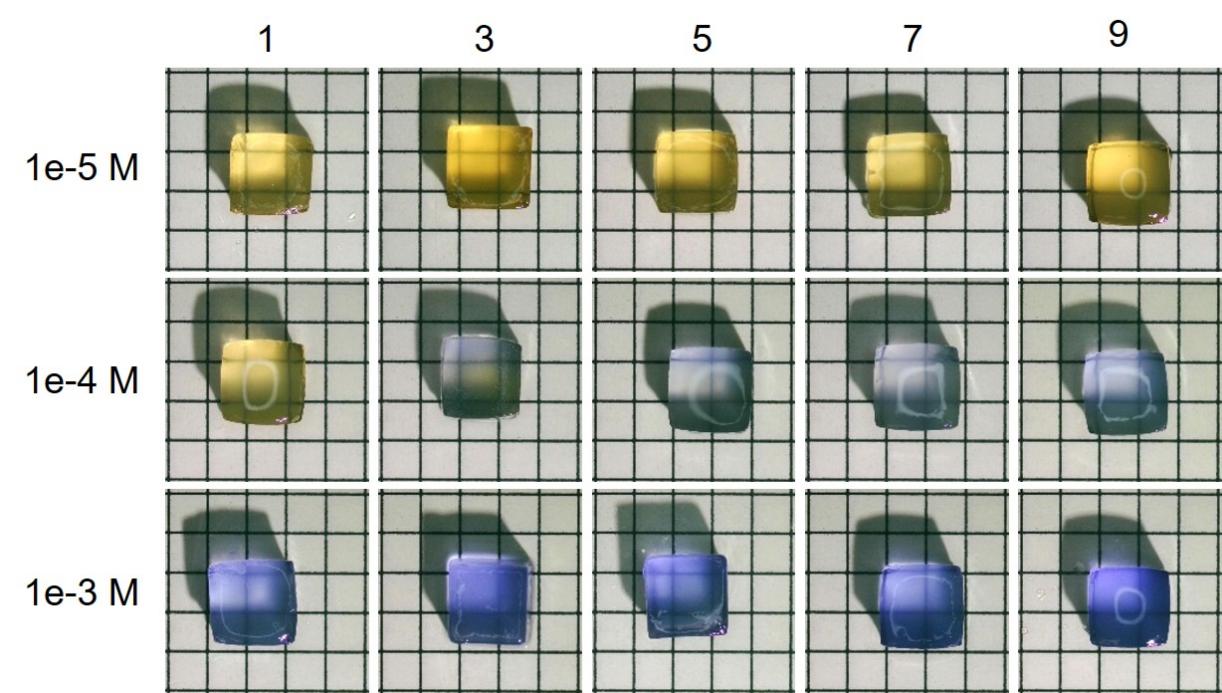


**Figure S14.** Colorimetric response of zincon-encapsulated 3D hydrogels to varying Zn^2+^ concentrations.


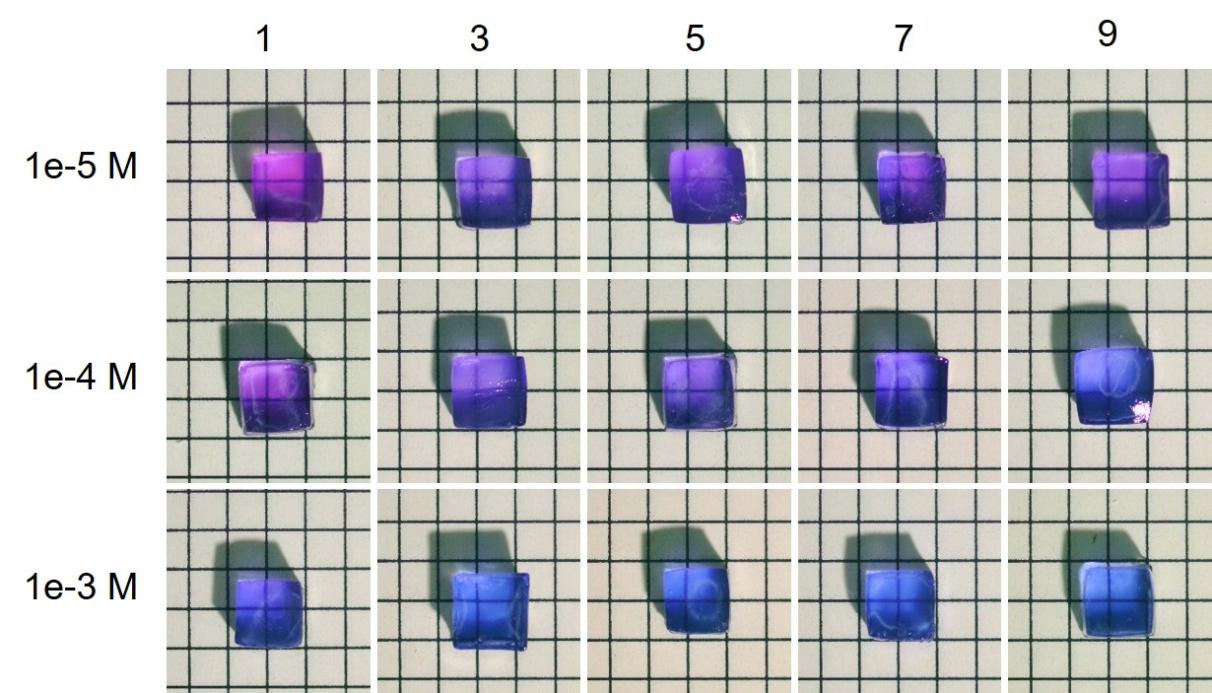


**Figure S15.** Colorimetric response of CPA-mA-encapsulated 3D hydrogels to varying Ca^2+^ concentrations.


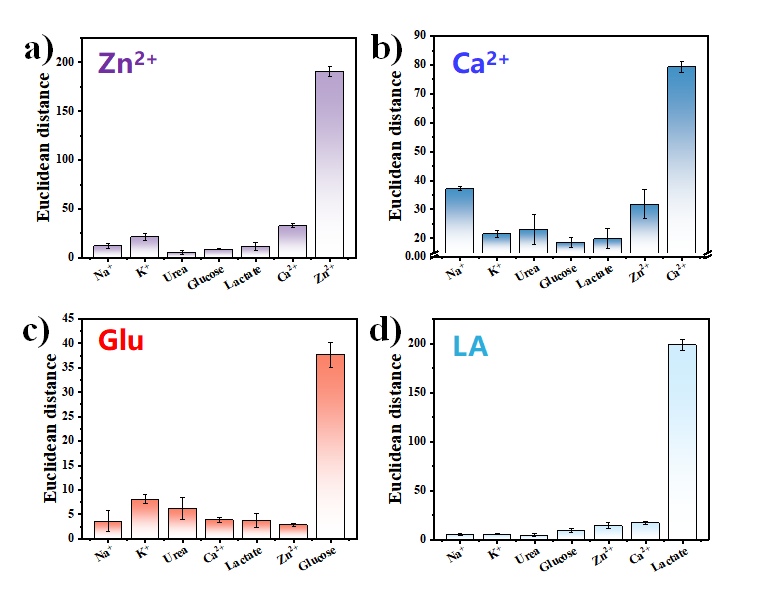


**Figure S16.** Selectivity of the a) Zn^2+^, b) Ca^2+^, c) glucose, and d) lactate colorimetric 3D colorimetric hydrogel patch(Euclidean distance =$\sqrt{\left( \text{R−}\text{R}_{\text{0}} \right)^{\text{2}}\text{+}\left( \text{B−}\text{B}_{\text{0}} \right)^{\text{2}}\text{+}\left( \text{G−}\text{G}_{\text{0}} \right)^{\text{2}}}$)

1. ***Hardware and software configuration.***

All programs were written in Python. The PIL library was used to process the image datasets, the Numpy library was used to process the pixel values into a matrix, and the OpenCV library was used to read the pixel values of the image. All computations and learning methods were complemented in Visual Studio Code (version 1.63.2) in Microsoft Windows 10 (x64), The CPU version was Intel Core i5-5200. All deep learning models (CNN, ANN) models were constructed by Pytorch. Machine learning models (DT, KNN, IR, NB, RF, SVM) were constructed by scikit-learn. XGBoost model was constructed by XGBoost library.

The classification model was constructed and optimized using a CNN-based deep learning algorithm. Although the original image resolution is 1000 × 1000 pixels, the code automatically resizes all input images to a standardized size of 100 × 100 pixels before feeding them into the network. The classifier employed cross-entropy as its loss function, with the Adam optimizer utilized for network optimization. Model performance was preliminarily evaluated and hyperparameters were refined by monitoring both the cross-entropy loss and prediction accuracy on the validation set. The optimized CNN classifier was ultimately configured with the following hyperparameters: 100 training epochs, a learning rate of 0.0001, and a consistent batch size of 64 for both training and validation sets.

For the regression task, we implemented two deep learning architectures (CNN and ANN) alongside six machine learning algorithms (Decision Tree, K-Nearest Neighbors, Linear Regression, Random Forest, Support Vector Machine, and XGBoost). The CNN architecture comprised an input layer, four convolutional layers with ReLU activation functions, fully-connected layers, and a single-neuron output layer for direct concentration value prediction. All input images were standardized to 100×100 pixel resolution during both training and testing phases. The model's optimization process employed Mean Squared Error (MSE) as the loss function with Adam optimizer. Model performance was rigorously evaluated using four quantitative metrics: coefficient of determination (R^2^), MSE, Root Mean Square Error (RMSE), and Mean Absolute Error (MAE), which simultaneously guided hyperparameter tuning. The optimal configuration was determined to be: 100 training epochs, batch size of 64, and learning rate of 0.0001, achieving balanced computational efficiency and predictive accuracy.

1. ***Construction of the dataset.***

We established 15 concentration gradients for each type of biomarker (Zn^2+^, Ca^2+^, glucose, and lactate). For each concentration gradient, we independently prepared 10 replicate samples. All samples underwent the entire process of indicator loading, sample addition, and chromogenic reaction independently, ensuring the complete independence of each replicate sample during preparation. To expand the dataset size for subsequent deep learning analysis and achieve effective data augmentation, we performed 15 imaging captures for each independent replicate sample by rotating them 90 degrees, flipping them over, and rotating them again. Therefore, the total number of images collected is calculated as follows: 4 types of biomarkers × 15 concentration gradients × 10 replicate samples × 15 images per sample = 9,000 images. The entire dataset for each substance was then completely and randomly shuffled and divided into training, validation, and test sets in an 8:1:1 ratio.

1. ***Confusion matrix of the Machine Learning classification model.***


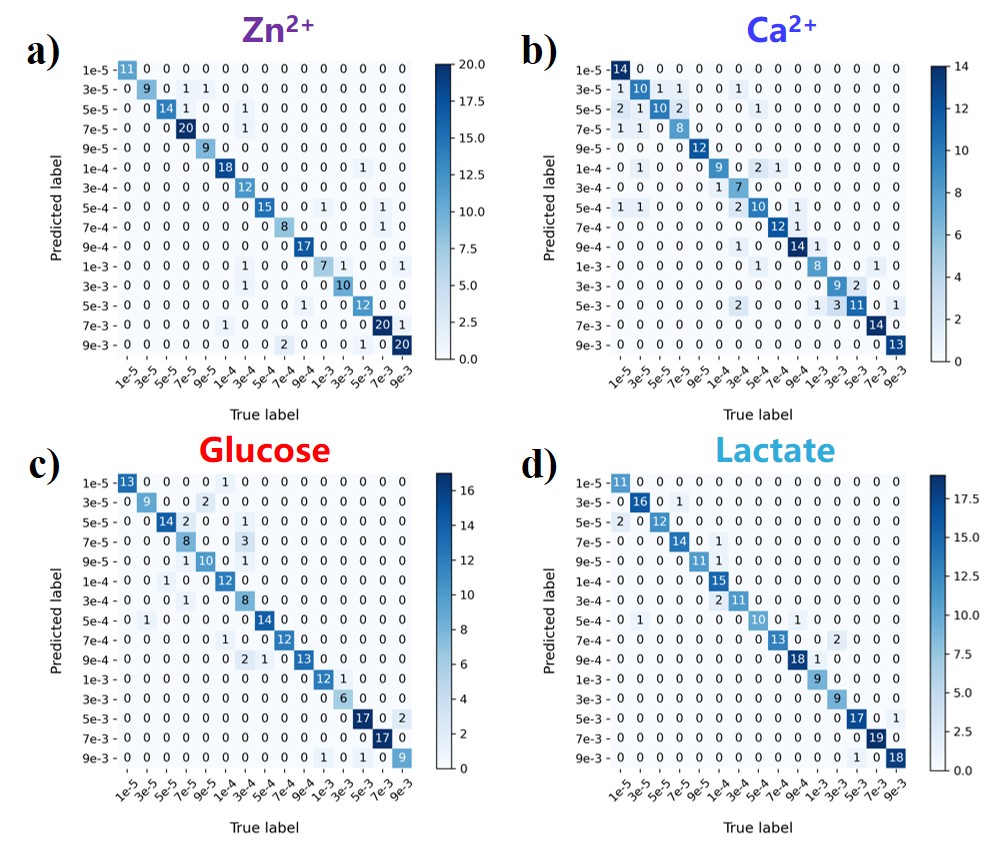


**Figure S17.** Confusion matrix of the DT classification model for a) zinc ions, b) calcium ions, c) glucose and d) lactate with varying concentrations.


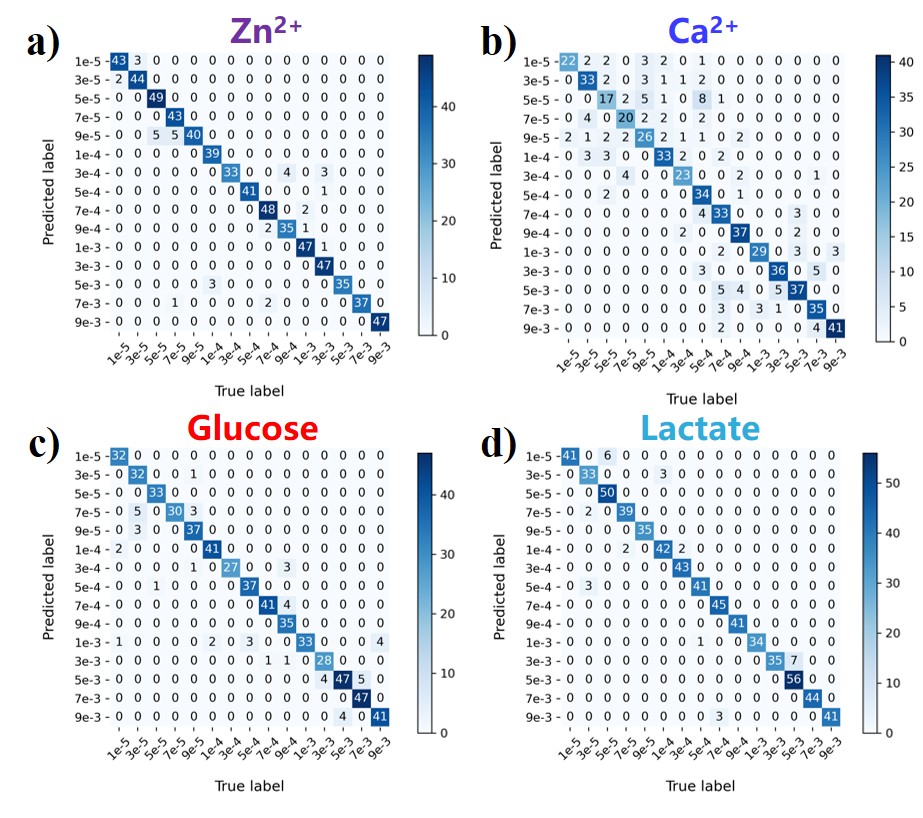


**Figure S18.** Confusion matrix of the KNN classification model for a) zinc ions, b) calcium ions, c) glucose and d) lactate with varying concentrations.


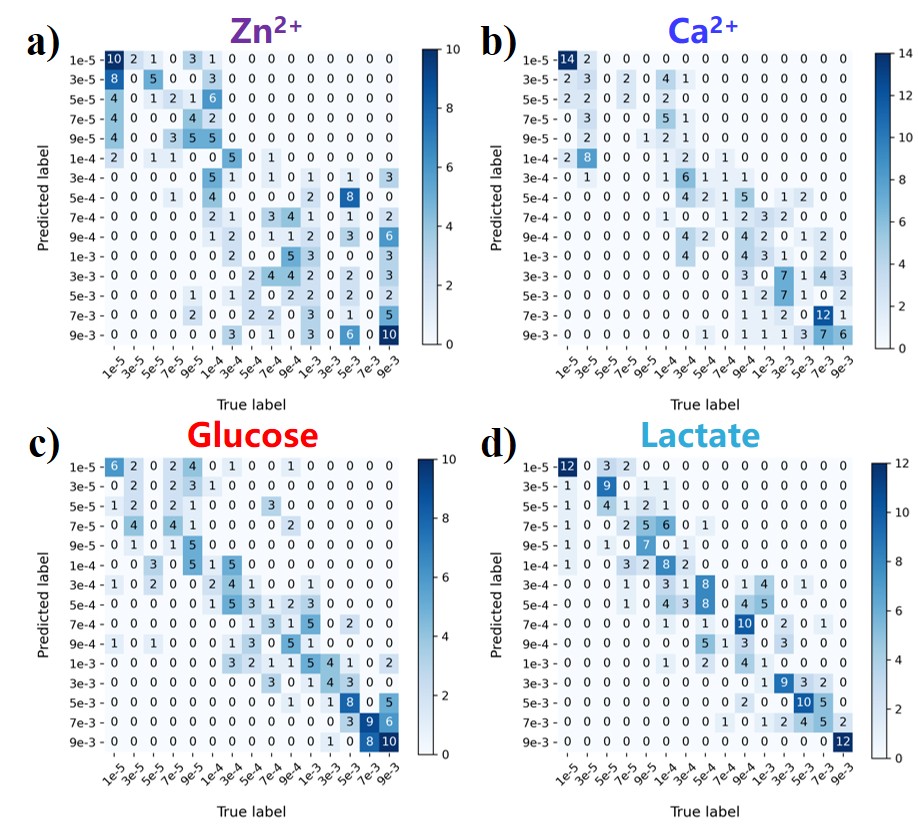


**Figure S19.** Confusion matrix of the IR classification model for a) zinc ions, b) calcium ions, c) glucose and d) lactate with varying concentrations.


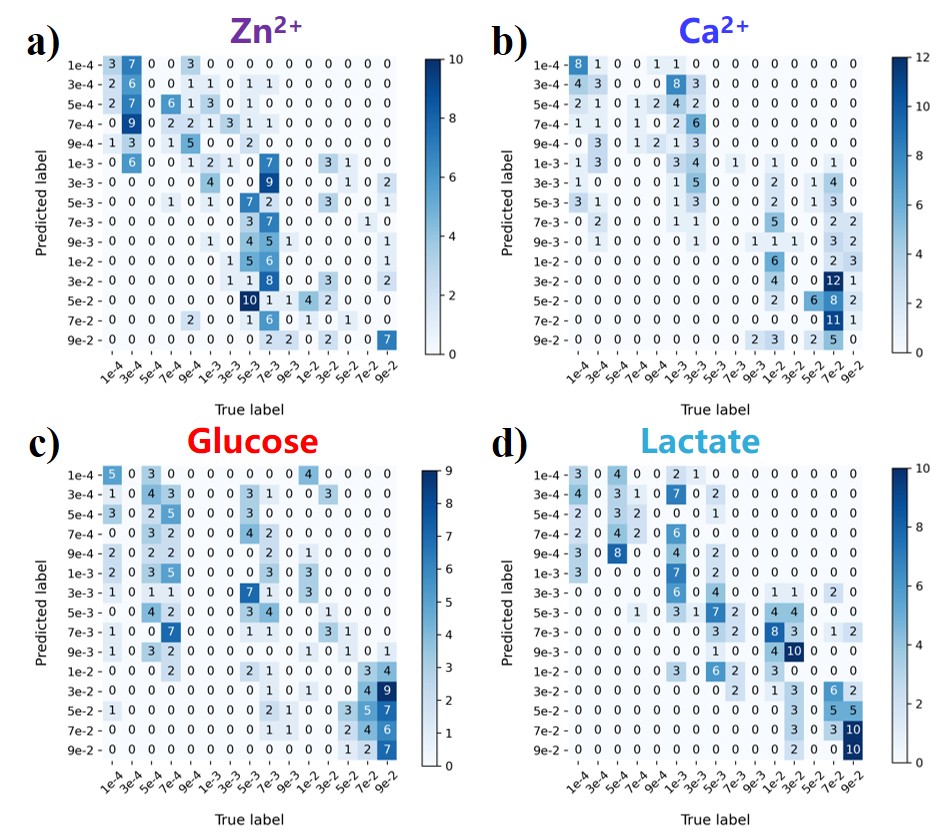


**Figure S20.** Confusion matrix of the NB classification model for a) zinc ions, b) calcium ions, c) glucose and d) lactate with varying concentrations.


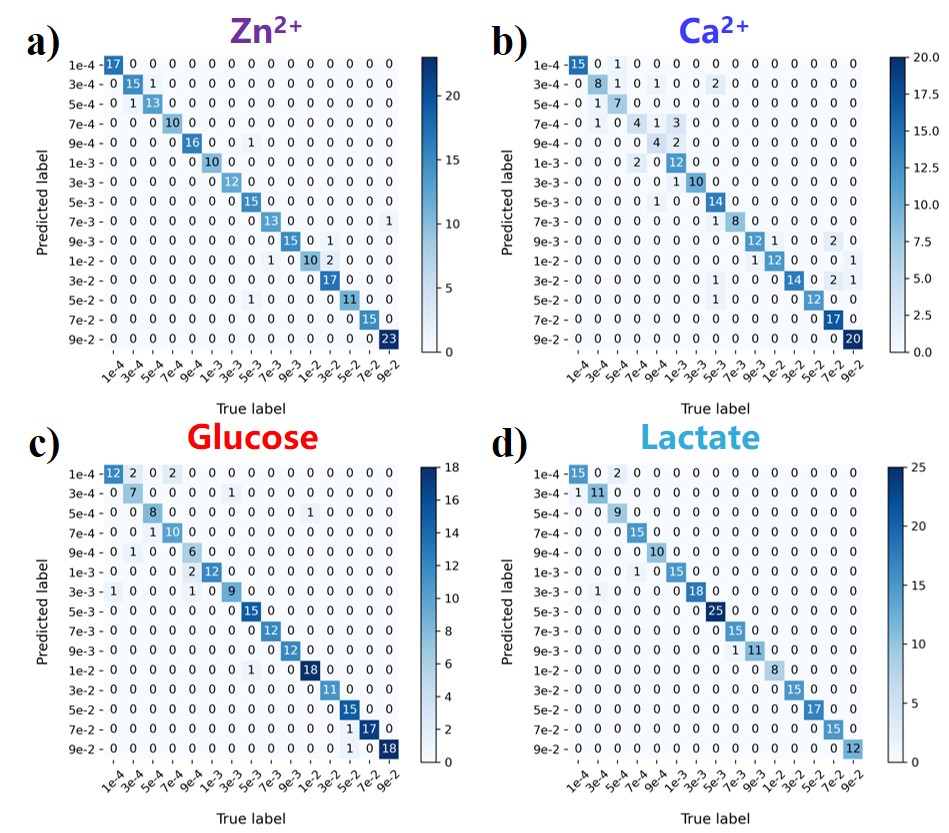


**Figure S21.** Confusion matrix of the RF classification model for a) zinc ions, b) calcium ions, c) glucose and d) lactate with varying concentrations.


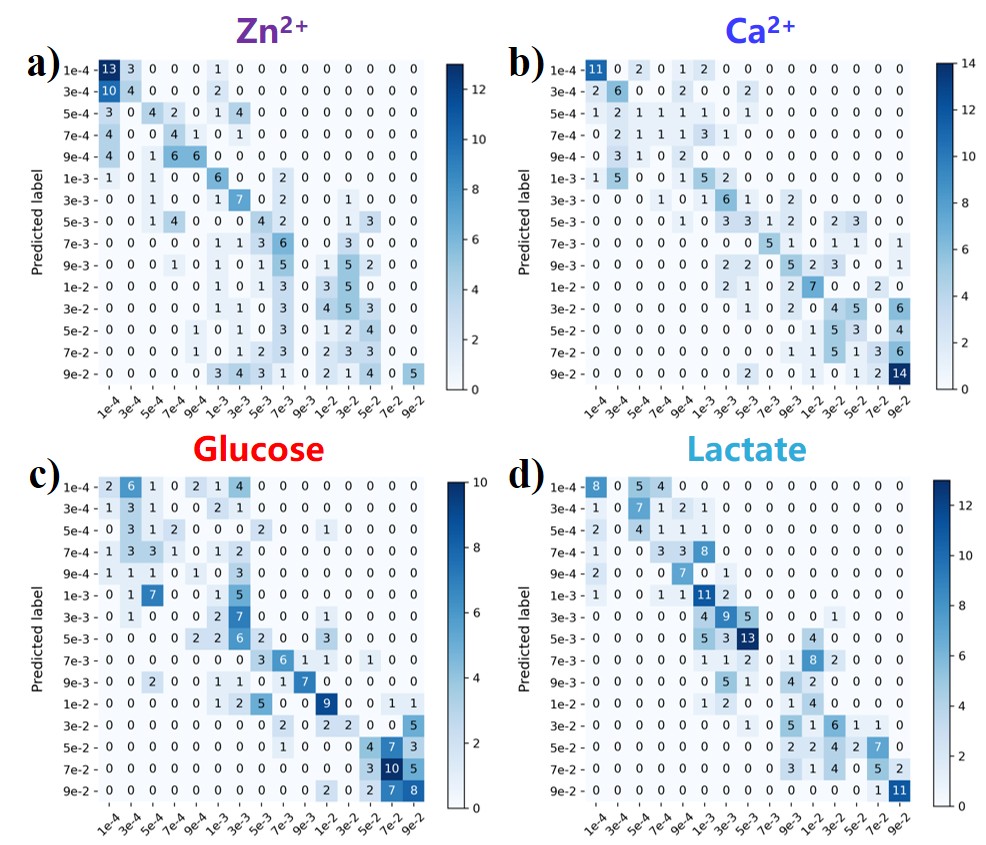


**Figure S22.** Confusion matrix of the SVM classification model for a) zinc ions, b) calcium ions, c) glucose and d) lactate with varying concentrations.


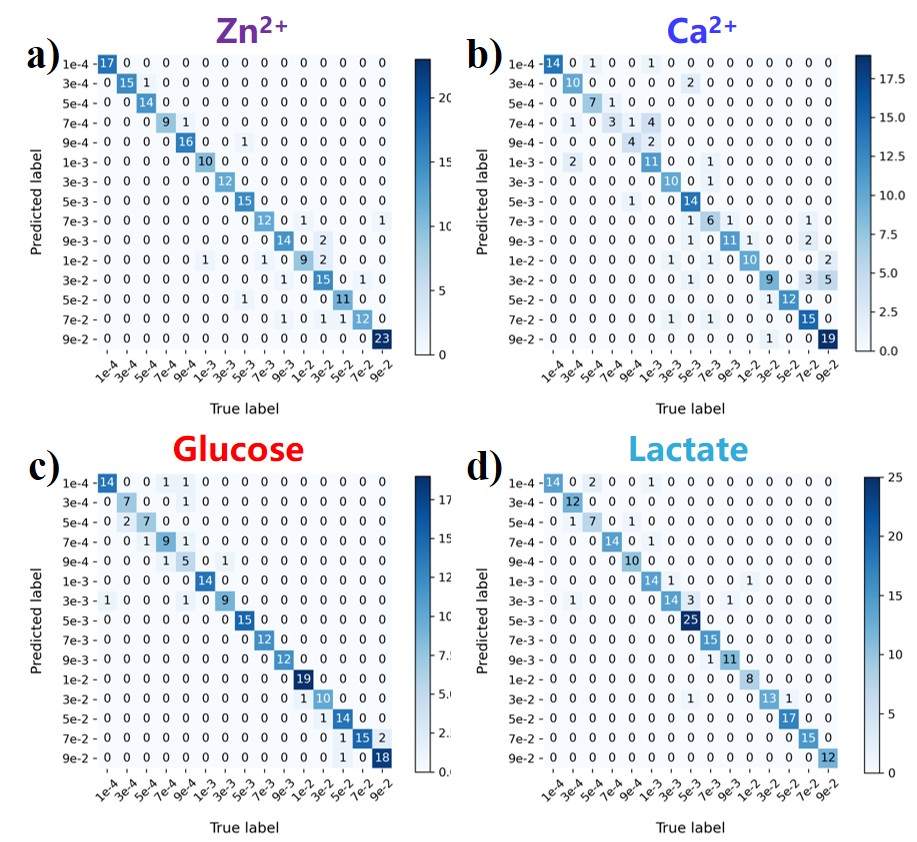


**Figure S23.** Confusion matrix of the XGB classification model for a) zinc ions, b) calcium ions, c) glucose and d) lactate with varying concentrations.

1. ***Confusion matrix of the CNN classification model.***


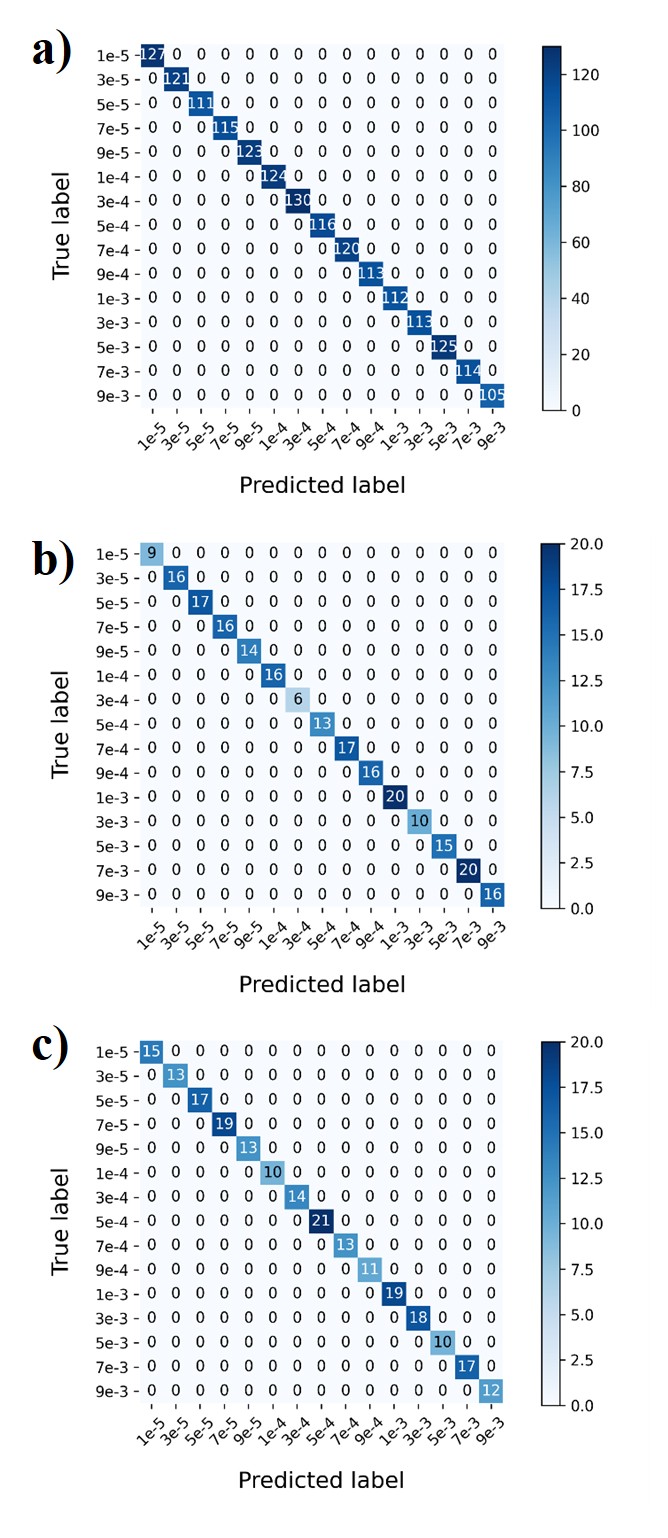


**Figure S24.**Confusion matrices of the CNN classification model for Zn^2+^ samples with varying concentrations on the a) training, b) validation, and c) test sets.


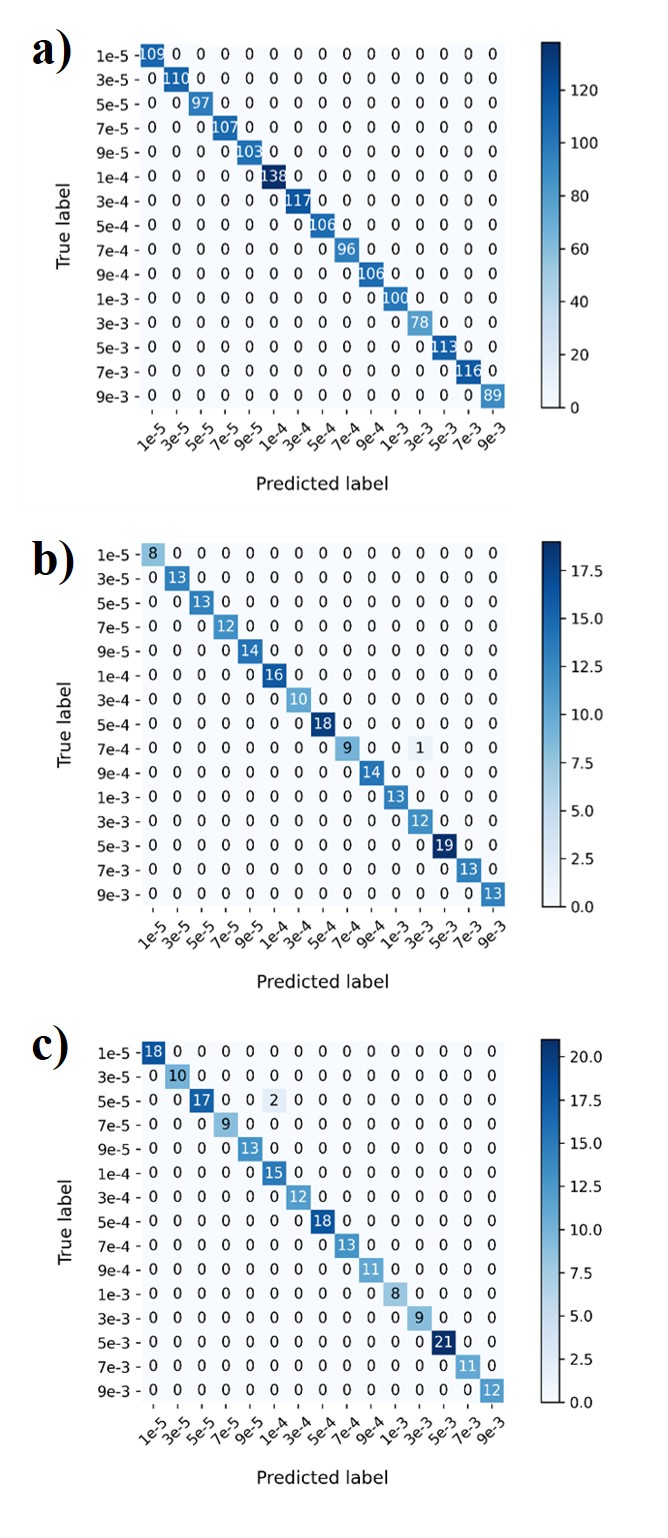


**Figure S25.** Confusion matrices of the CNN classification model for Ca^2+^ samples with varying concentrations on the a) training, b) validation, and c) test sets.


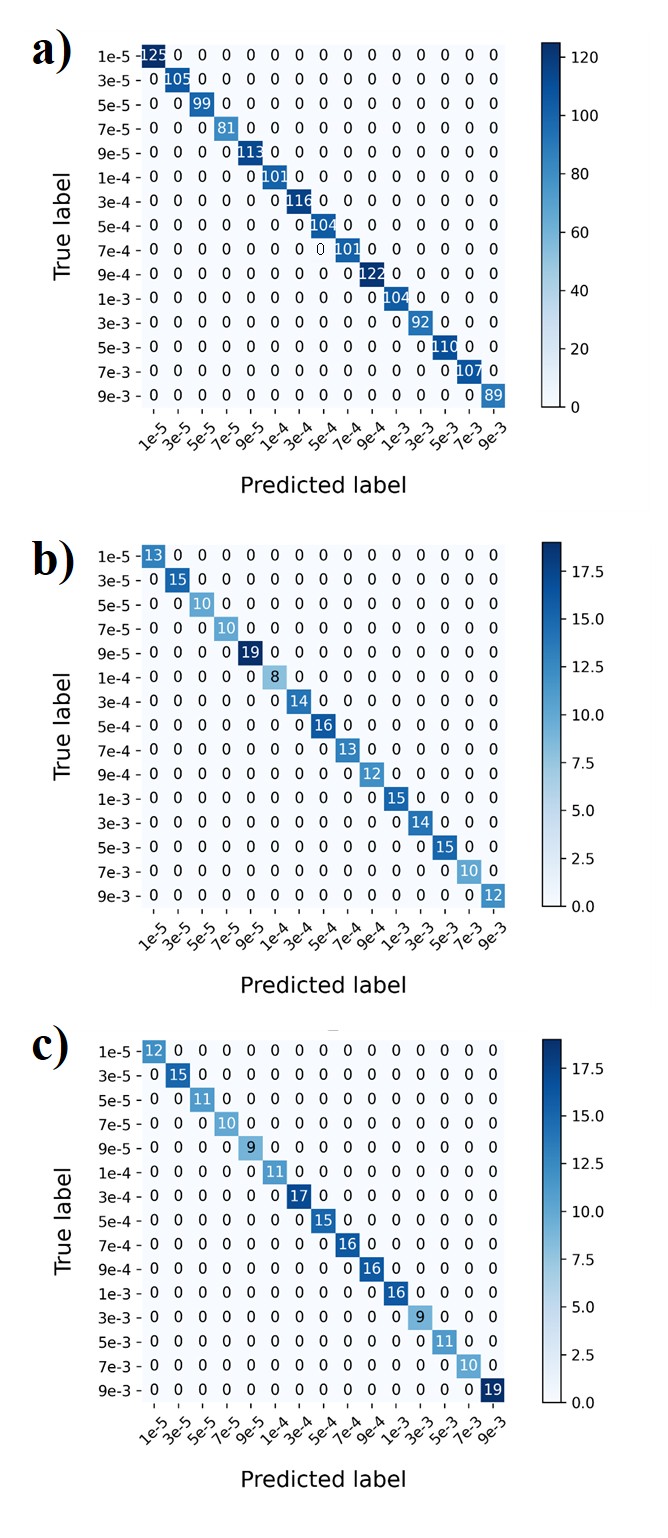


**Figure S26.**Confusion matrices of the CNN classification model for glucose samples with varying concentrations on the a) training, b) validation, and c) test sets.


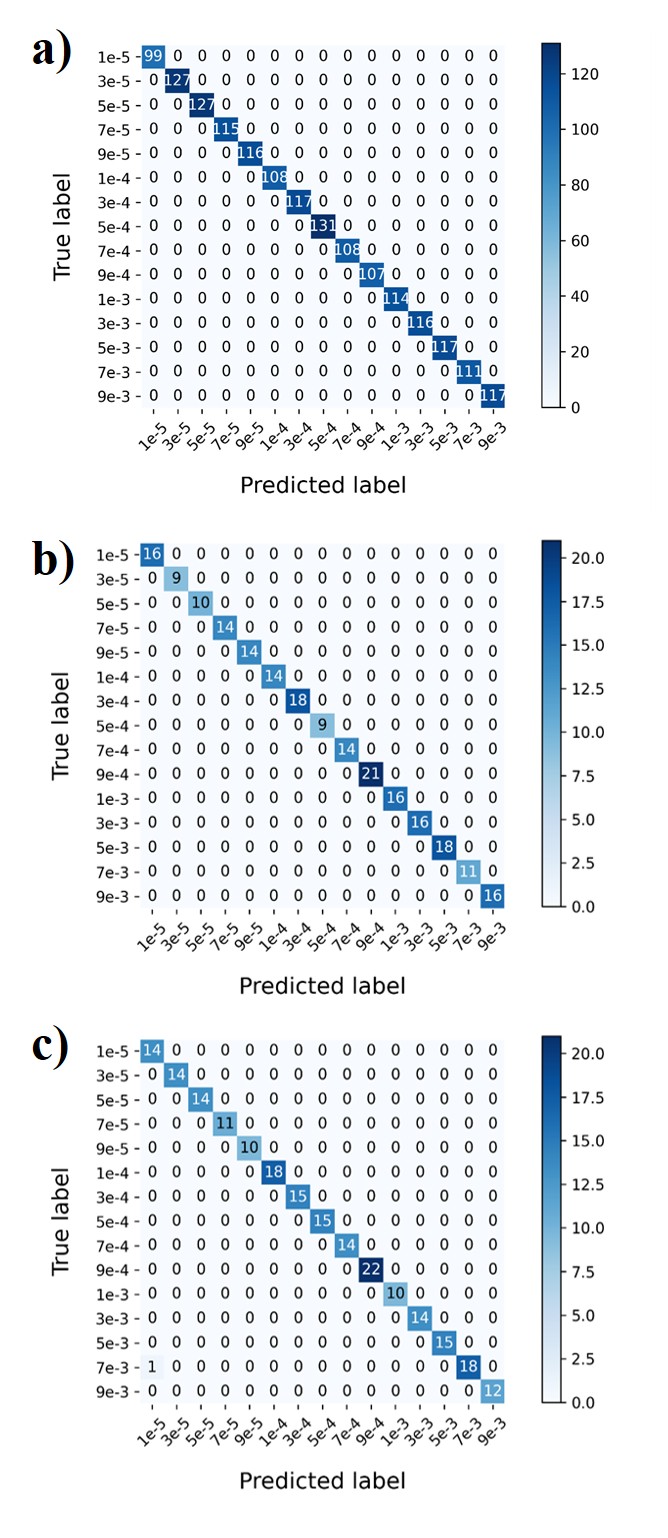


**Figure S27.**Confusion matrices of the CNN classification model for lactate samples with varying concentrations on the a) training, b) validation, and c) test sets.

1. ***Machine Learning and Deep Learning regression results.***


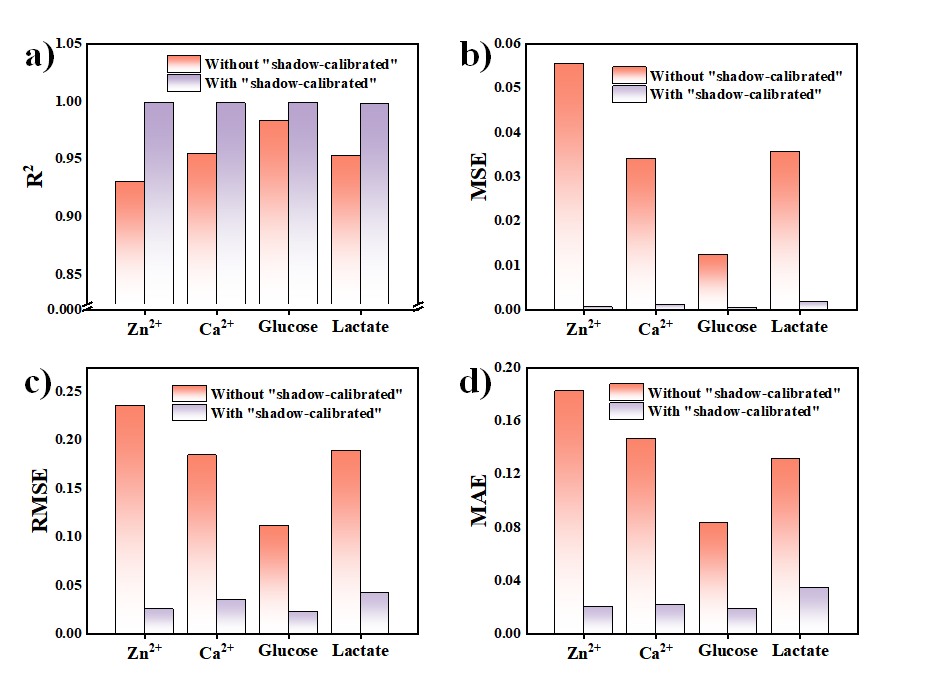


**Figure S28.** Model evaluation metrics without and with the shadow-calibrated strategy a) R², b) MSE, c) RMSE, d) MAE (all error metrics were calculated in logarithm of concentration space).


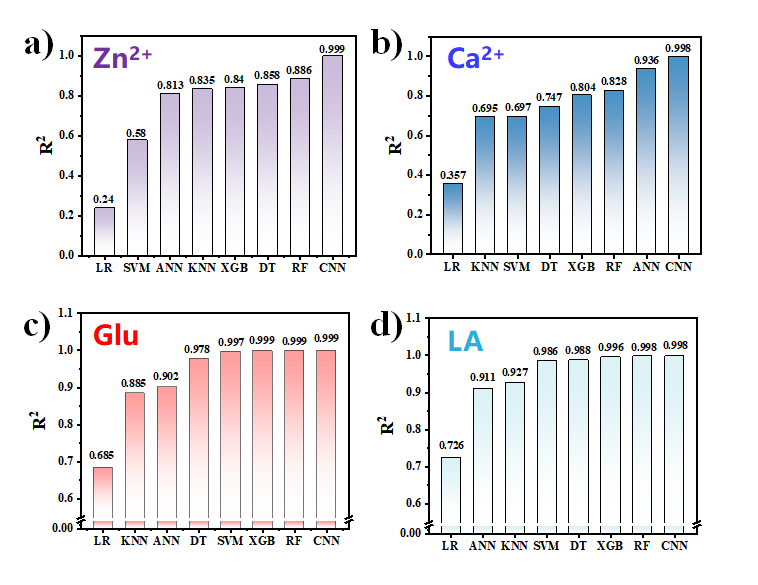


**Figure S29.** Machine Learning and Deep Learning regression results of a) zinc ions, b) calcium ions, c) glucose and d) lactate.

1. ***Machine Learning and Deep Learning results for actual artificial sweat.***


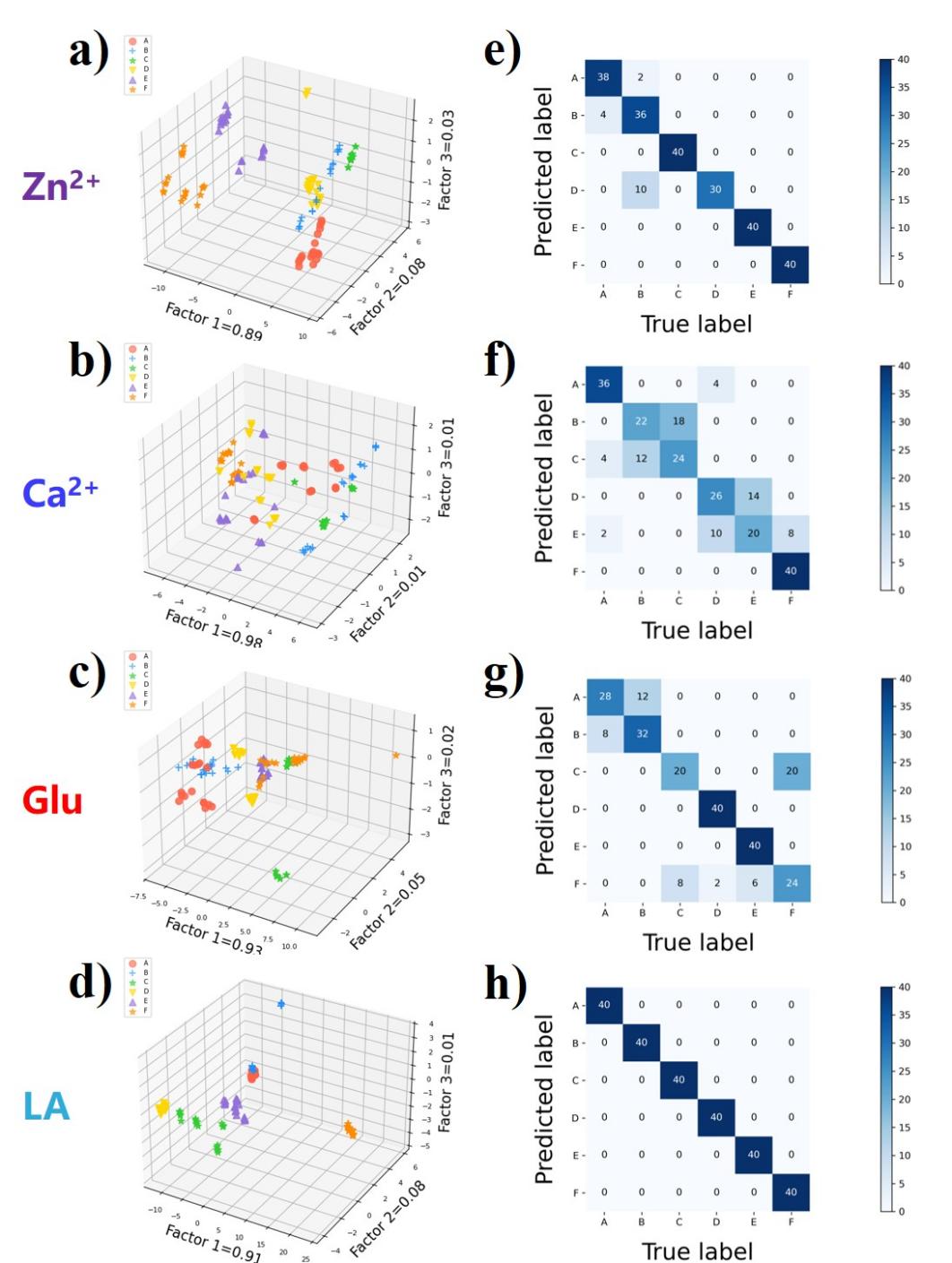


**Figure S30.** a-d) LDA and corresponding, e-h) confusion matrix of zinc ions, calcium ions, glucose, and lactate in artificial sweat.


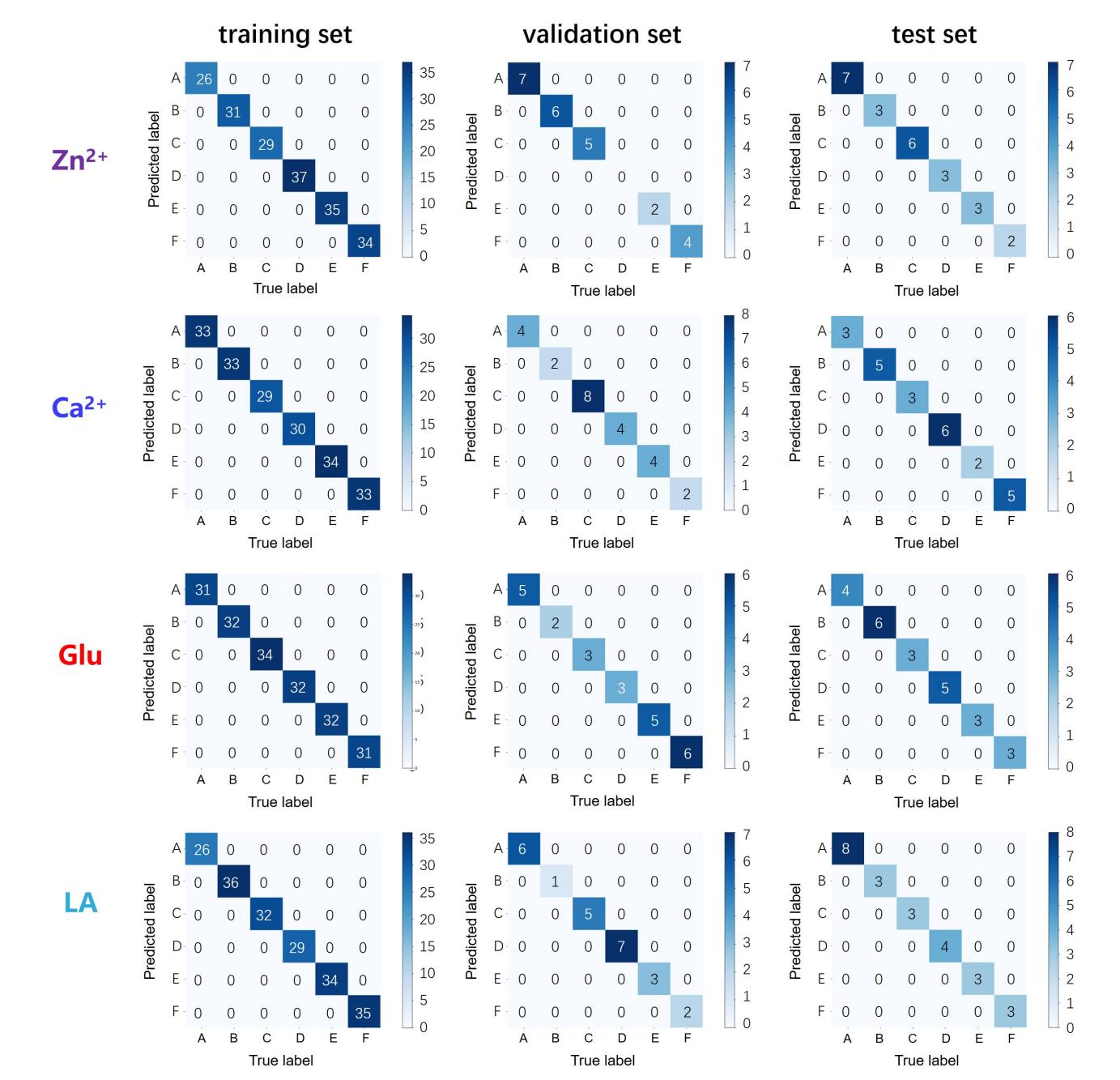


**Figure S31.** Results of the CNN classification model for zinc ions in six types of actual artificial sweat on training, validation and test sets.

1. ***The CAM heatmaps of the CNN regression analysis.***


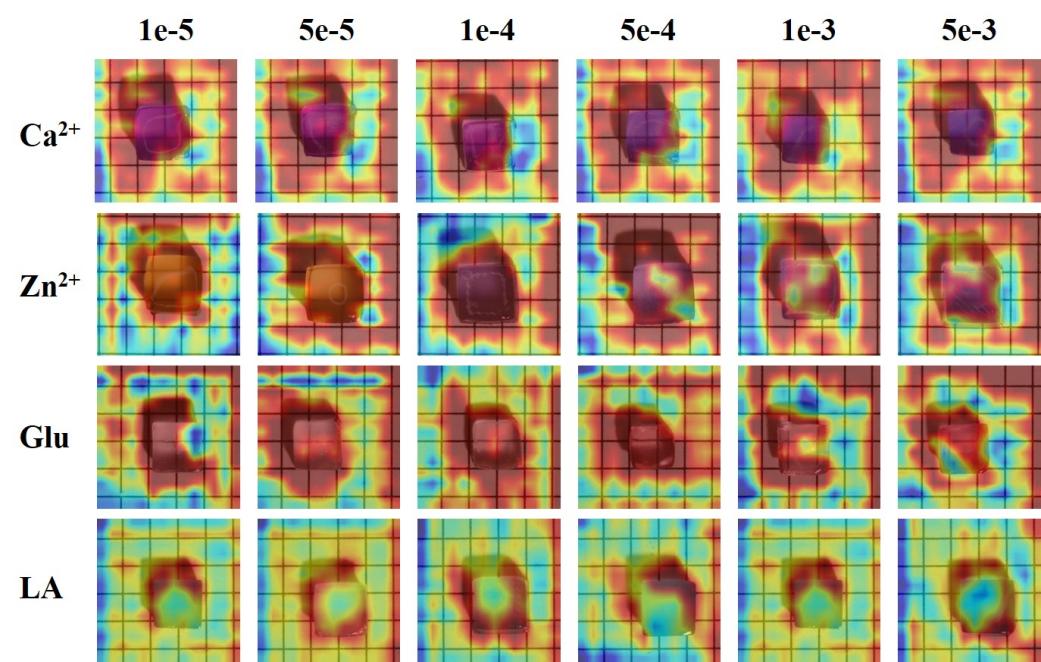


**Figure S32.** The CAM heatmaps of the CNN regression analysis of four sweat biomarkers.

1. ***Parameters of classification and regression models.***

**Table S1.** Parameters of ML classification models.

| **Algorithm** | **Parameters** |
| --- | --- |
| LDA | (solver='svd', shrinkage=None, priors=None, n_components=3, store_covariance=False, tol=0.0001) |
| Decision Trees | (criterion = 'gini', splitter = 'best', max_depth = None, min_samples_leaf = 1, min_samples_split = 2) |
| KNN | (n_neighbors = 1, weights = 'uniform', algorithm = 'auto', leaf_size = 30, p = 2, metric = 'minkowski') |
| Logistic Regression | (solver = 'sag', penalty = 'l2', C = 13.01, max_iter = 60) |
| Naive Bayes | (num_round = 300, criterion = 'gini', gamma = 1.77, subsample = 0.75) |
| Random Forest | (criterion = 'gini', n_estimators = 41, max_depth = 15, min_samples_leaf = 2, min_samples_split = 2) |
| SVM | (kernel = 'rbf', gamma = 1, C = 0.622) |
| XGBoost | (num_round = 90, eta = 0.25, max_dept = 6, subsample = 0.55) |

**Table S2.** The architecture of the CNN classification model.

| **Layer** | **Layer (type)** | **Output Shape** |
| --- | --- | --- |
| 1 | Input layer | (None, 100, 100, 3) |
| 2 | Convolution layer | (None, 98, 98, 32) |
| 3 | Max pooling layer | (None, 49, 49, 32) |
| 4 | Convolution layer | (None, 47, 47, 64) |
| 5 | Max pooling layer | (None, 23, 23, 64) |
| 6 | Convolution layer | (None, 21, 21, 128) |
| 7 | Max pooling layer | (None, 10, 10, 128) |
| 8 | Convolution layer | (None, 8, 8, 128) |
| 9 | Max pooling layer | (None, 4, 4, 128) |
| 10 | Flatten layer | (None, 2048) |
| 11 | Fully-connected layer | (None, 128) |
| 12 | Dropout layer | (None, 128) |
| 13 | Fully-connected layer | (None, 20 or 6) |
| 14 | Output | (None, 20 or 6) |

**Table S3.** The architecture of the CNN quantification model.

| **Layer** | **Layer (type)** | **Output Shape** |
| --- | --- | --- |
| 1 | Input layer | (None, 100, 100, 3) |
| 2 | Convolution layer | (None, 98, 98, 32) |
| 3 | Max pooling layer | (None, 49, 49, 32) |
| 4 | Convolution layer | (None, 47, 47, 64) |
| 5 | Max pooling layer | (None, 23, 23, 64) |
| 6 | Convolution layer | (None, 21, 21, 128) |
| 7 | Max pooling layer | (None, 10, 10, 128) |
| 8 | Convolution layer | (None, 8, 8, 128) |
| 9 | Max pooling layer | (None, 4, 4, 128) |
| 10 | Flatten layer | (None, 2048) |
| 11 | Fully-connected layer | (None, 128) |
| 12 | Dropout layer | (None, 128) |
| 13 | Fully-connected layer | (None, 1) |
| 14 | Output | (None, 1) |

**Table S4.** Parameters of ML quantification models.

| **Algorithm** | **Parameters** |
| --- | --- |
| Decision Trees | (criterion = 'poisson', splitter = 'best', max_depth = 23, min_samples_leaf = 1, min_samples_split = 2) |
| KNN | (n_neighbors = 10, weights = 'uniform', algorithm = 'auto', leaf_size = 30, p = 2, metric = 'minkowski') |
| Logistic Regression | (solver = 'sag', penalty = 'l2', C = 14, max_iter = 560) |
| Random Forest | (criterion = 'absolute_error', n_estimators = 41, max_depth = 23, min_samples_leaf = 1, min_samples_split = 2) |
| SVM | (kernel = 'rbf', gamma = 2.88, C = 100) |
| XGBoost | (num_round = 100, eta = 0.4, max_dept = 7, subsample = 0.65) |

**Table S5.** The architecture of the ANN quantification model.

| **Layer** | **Layer (type)** | **Output Shape** |
| --- | --- | --- |
| 1 | Input layer | (None, 18) |
| 2 | Fully-connected layer | (None, 64) |
| 3 | Fully-connected layer | (None, 256) |
| 4 | Fully-connected layer | (None, 128) |
| 5 | Dropout layer | (None, 64) |
| 6 | Fully-connected layer | (None, 1) |
| 7 | Output | (None, 1) |

**Table S6.** Quantification Performance of Zn^2+^ Across Three Sub-Datasets Using Different Quantification Models.

**Table S7.** Quantification Performance of Ca^2+^ Across Three Sub-Datasets Using Different Quantification Models.

**Table S8.** Quantification Performance of Glucose Across Three Sub-Datasets Using Different Quantification Models.

**Table S9.** Quantification Performance of Lactate Across Three Sub-Datasets Using Different Quantification Mode.
